# Plastidic membrane lipids are oxidized by a lipoxygenase in *Lobosphaera incisa*

**DOI:** 10.3389/fpls.2022.1102215

**Published:** 2022-12-22

**Authors:** Benjamin Djian, Kirstin Feussner, Cornelia Herrfurth, Krzysztof Zienkiewicz, Ellen Hornung, Ivo Feussner

**Affiliations:** ^1^ Albrecht-von-Haller-Institute for Plant Sciences, Department of Plant Biochemistry, University of Goettingen, Goettingen, Germany; ^2^ Goettingen Center for Molecular Biosciences (GZMB), Service Unit for Metabolomics and Lipidomics, University of Goettingen, Goettingen, Germany; ^3^ Goettingen Center for Molecular Biosciences (GZMB), Department of Plant Biochemistry, University of Goettingen, Goettingen, Germany

**Keywords:** chlorophyte, galactolipid, lipid peroxidation, lipoxygenase, metabolite fingerprinting, oxylipins, polyunsaturated fatty acids

## Abstract

Green microalgae can accumulate neutral lipids, as part of a general lipid remodeling mechanism under stress such as nitrogen starvation. *Lobosphaera incisa* is of special interest because of its unique TAG acyl chain composition, especially 20:4 (n-6) can reach up to 21% of dry weight after nitrogen starvation. In order to identify factors that may influence the accumulation of polyunsaturated fatty acids (PUFAs), we identified recently a linoleate 13-lipoxygenase (LiLOX). It shares highest identity with plastidic enzymes from vascular plants and is induced upon nitrogen starvation. Here, we confirmed the localization of LiLOX in the stroma of plastids *via* transient expression in epithelial onion cells. In order to further characterize this enzyme, we focused on the identification of the endogenous substrate of LiLOX. In this regard, an *ex vivo* enzymatic assay, coupled with non-targeted analysis *via* mass spectrometry allowed the identification of MGDG, DGDG and PC as three substrate candidates, later confirmed *via in vitro* assays. Further investigation revealed that LiLOX has preferences towards the lipid class MGDG, which seems in agreement with its localization in the galactolipid rich plastid. Altogether, this study shows the first characterization of plastidic LOX from green algae, showing preference for MGDGs. However, lipidomics analysis did neither reveal an endogenous LiLOX product nor the final end product of MGDG oxidation. Nevertheless, the latter is a key to understanding the role of this enzyme and since its expression is highest during the degradation of the plastidic membrane, it is tempting to assume its involvement in this process.

## Introduction

Lipoxygenases (LOXs) are non-heme iron enzymes intensively studied, known to catalyze the oxidation of polyunsaturated fatty acids (PUFAs) such as 18:2 (n-6) and 20:4 (n-6) (Brash, 1999; Liavonchanka and Feussner, 2006). LOXs are known to play important role in the regulation of essential signaling pathways in vascularized organisms. In mammals for instance, LOXs are known to regulate inflammatory responses, cancer, developmental processes, and blood pressure, cell differentiation (Kühn et al., 2015). In vascular plants, LOXs were found to be essential for the formation of the phytohormone jasmonoyl isoleucine and are therefore crucial for male fertility, wound response and defense against insects and pathogens among others (Wasternack and Feussner, 2018; Viswanath et al., 2020). LOX products were further found to be precursors of green leaf volatiles and divinyl ethers which are involved in defense against herbivores and pathogens (Christensen and Kolomiets, 2011; Oliw, 2022). In addition to free PUFAs, some LOXs were also found to accept esterified acyl chains as substrate, for example, the rabbit reticulocyte LOX, cucumber lipid body LBLOX, and *Pseudomonas aeruginosa* LOX (PaLOX) (Schewe et al., 1986; Feussner et al., 1998; Banthiya et al., 2015). This opened the hypothesis that LOX could play a role in membrane degradation as well as LB degradation (Schewe et al., 1986; Feussner et al., 1995; van Leyen et al., 1998; Aldrovandi et al., 2018).

In the last decades, LOXs were also discovered in cyanobacteria and green microalgae (Lang and Feussner, 2007; Andreou et al., 2008; Andreou et al., 2010; Newie et al., 2016; Djian et al., 2019). Notably, LOX transcripts from green microalgae were reported to be upregulated under different types of stress conditions (Légeret et al., 2016; Prasad et al., 2016; Kokabi et al., 2020). The stress conditions in plants and microalgae being responsible for major lipid remodeling were intensively studied, namely accumulation of neutral lipids and reduction of chloroplast membranes (Zienkiewicz et al., 2016). The reduction of the chloroplast membranes was observed by fluorescent and electron microscopy as well as by lipidomics analysis of monogalactosyldiacylglycerol (MGDG) and digalactosyldiacylglycerol (DGDG) (Merzlyak et al., 2007; Kokabi et al., 2020). These two lipid classes represent the main lipid constituents of the plastidial membranes. For instance, they represent about 75% of all lipid species found in thylakoid membranes and in the inner envelope of tobacco leaves (Block et al., 1983; Dorne et al., 1990). The reduction of chloroplast membranes and the accumulation of neutral lipids during stress conditions was suggested to have two roles: (i) Reconversion of thylakoid membrane lipids into storage lipids could serve as energy source (Feussner and Wasternack, 1998; Moellering et al., 2010; Légeret et al., 2016). (ii) Under nitrogen starvation, the electrons flow starting from the water splitting, reducing NADP^+^ into NADPH, cannot be used for the *de novo* synthesis of DNA and proteins. Since the ratio of electron donor/acceptor (NADPH/NADP^+^) is decreasing, electrons flowing at the photosystem I might cause reduction of molecular oxygen inside the thylakoid lumen, forming reactive oxygen species. It was proposed that the *de novo* synthesis of free fatty acids, rather than the acyl chain recycling, plays a vital role as electron sink (Fan et al., 2011; Fan et al., 2012; Li et al., 2012).

The green microalga *Lobosphaera incisa* (*L. incisa*, formerly referred to as *Parietochloris incisa* or *Myrmecia incisa*), was reported to accumulate up to 77% of all acyl chains in TAG during the stationary growth phase. Moreover, TAG from *L. incisa* was reported to be composed of 47% 20:4 (n-6) (Bigogno et al., 2002). As it is an oleaginous green alga, the accumulation of starch under stress is limited, and lipid content was reported to reach 35% of dry weight after nitrogen starvation, which consists of 21% 20:4 (n-6) acyl chains alone. Due to this latter property this strain of microalgae is becoming an important model for its capacity to accumulate very long chain PUFAs under stress. Interestingly, in the transcriptomic data of *L. incisa*, a LOX was discovered and found to be active through heterologous expression in *E. coli* (Siegler et al., 2017; Djian et al., 2019; Kokabi et al., 2020). This LiLOX was also shown to have highest homology with plastidial LOX and is therefore suspected to localize to chloroplast. Furthermore, transcripts of LiLOX were found to be 13 fold upregulated upon nitrogen starvation (Kokabi et al., 2020; Kumari et al., 2020). Therefore, this study aims to confirm the endogenous subcellular localization of LiLOX and to identify its endogenous substrate(s) and allowing to generate a hypothesis about its native role under stress conditions.

## Materials and methods

### Chemicals

Propan-2-ol was obtained from Fisher Scientific GmbH (Schwerte, Germany). All additional chemicals were obtained from Carl Roth (Karlsruhe, Germany). Restriction enzymes were all obtained from MBI Fermentas (Thermo Fisher Scientific, Waltham, MA, USA) and PUFAs substrates as well as standards (hydroxy octadecadienoic acid: HODE, hydroxy octadecatrienoic acid: HOTE and hydroxy eicosatetraenoic acid: HETE) were purchased from Cayman Chemical Company (Ann Arbor, MI, USA). 18:3 (n-3)/18:3 (n-3)-PC (36:6-PC) was obtained from Larodan AB (Solna, Sweden).

### Algal growth conditions and cDNA synthesis

*L. incisa* strain SAG 2468 was kindly provided by Prof Inna Khozin-Goldberg, Ben-Gurion University of the Negev, Israel. *L. incisa* cells were grown under constant 25 °C, in a maximum volume of 300 mL BG11 medium inside glass columns (Stanier et al., 1971). Light of 190 µmol photons m^-2^ s^-1^ was provided constantly to the cells. The only source of carbon available to the algae was carbon dioxide (1%) provided by a constant gas flow, also used to agitate the cells to homogeny in the medium. Total RNA of *L. incisa* were obtained from 20 mg of dry cells using TRIzol (Invitrogen, Waltham, MA, USA). cDNA was obtained *via* RevertAid RT (Thermo Fisher Scientific, Waltham, MA, USA), using oligo(dt)18 as primers.

### qRT PCR

Total cDNA freshly synthetized was used as material for the qRT-PCR. The qRT-PCR reaction mixture was carried out using Takyon No Rox SYBR MasterMix dTTP Blue (Eurogentec, Seraing, Belgium). The PCR was performed in a thermocycler iQ5 qPCR cycler (Bio-Rad Laboratories GmbH, München, Germany). Data interpretation was achieve with the iQ5 software (BioRad Laboratories GmbH, München, Germany). The protein phosphatase 2A gene (PP2A) was used as a reference gene in *Arabidopsis thaliana*, as formerly described (Czechowski et al., 2005). The Ribosomal Protein S21 (RPS21) was chosen as a reference gene in *L. incisa*.

### Cloning

RACE-PCR was performed using the SMARTer^®^ RACE 5’/3’ Kit (Takara Bio Inc., Kusatsu, Japan) according to the manufacturer’s instructions. Inner primers: LiLOX Forward GGCATCGGCGCGTGAGGCAG, Reverse GACTACCCCTATGCAGCCGACG. LiLox was amplified using the following primers Forward: GAATTCGACAGCGTGCTTCCCCATGGC Reverse: TTGCGGCCGCTTACATTGAGACGCTGGTGGGAT. The PCR reaction has been performed with Phusion High Fidelity DNA polymerase (New England Biolabs Inc., Ipswich, MA, USA) and cloned in pET28a (Merck) using pJET cloning vector (Thermo Fisher Scientific, Waltham, MA, USA). The integrity of cloned vectors was verified by DNA sequencing (GATC Biotech AG, Ebersberg, Gemany).

Site directed mutagenesis was performed *via* PCR with Pfu polymerase (Thermo Fisher Scientific, Waltham, MA, USA) using the following primers: N687A-Forward CTCAACGTGAACTCCGCCGCCCGCCA. N687A-Reverse CAGCTGCTGGCGGGCGGCGGAGTTCA. N687R-Forward CTCAACGTGAACTCCAGGGCCCGCCA. N687R-Reverse CAGCTGCTGGCGGGCCCTGGAGTTCA. Q690A-Forward AACTCCAACGCCCGCGCGCAGCTGATT. Q690A-Reverse TGCATTAATCAGCTGCGCGCGGGCGTT. Q690R-Forward AACTCCAACGCCCGCCGGCAGCTGATT. Q690R-Reverse TGCATTAATCAGCTGCCGGCGGGCGT. Q691A-Forward TCCAACGCCCGCCAGGCGCTGATTAAT. Q691A-Reverse ACCTGCATTAATCAGCGCCTGGCGGGC. Q691R-Forward TCCAACGCCCGCCAGCGGCTGATTAAT. Q691R-Reverse ACCTGCATTAATCAGCCGCTGGCGGGC.

### Kinetic assays

Lipoxygenase activity was measured by spectrophotometric assays (CARY 100 Bio, Varian, Palo Alto, CA, USA) following the increase of absorbance at the wavelength 234 nm (ϵ = 2.5 x 10^4^ M^-1^ cm^-1^). All kinetic reactions were measured in 1 mL of 20 mM Bis-TRIS propane buffer at 30°C. Unless otherwise specified, reactions were started by adding 1 µg of LiLOX. Complex lipids, MGDG, DGDG or PC were solubilized in the reaction buffer at the final concentration of 100 µM. In order to achieve solubility of complex lipids, the reaction mixture included either 0.1% (w/v) sodium deoxycholate or 10% (v/v) ethanol depending on the following analysis. Velocity of LiLOX WT was measured with complex lipids mixture extracted from *L. incisa*. Velocity of LiLOX mutants (N687A, N687R, Q690A, Q690R, Q691A and Q691R) was measured with 36:6-MGDG (18:3 (n-3)/18:3 (n-3)-MGDG) extracted from pea leaves, and 36:6-PC (18:3 (n-3)/18:3 (n-3)-PC) purchased from Larodan AB (Solna, Sweden).

### Reduction and extraction of complex lipids oxidation products

In order to extract the oxidation products of complex lipids from the reaction mixture, a volume of methanol and a volume of chloroform were added to the aqueous buffer, for a final ratio of water:methanol:chloroform 1:1:1 (v/v/v). After vigorous vortexing for 30 seconds, and centrifugation for 1 minute, the hydrophobic phase was transferred in a glass tube, dried and solubilized in 200 µL methanol. When specified, oxidation products were reduced prior to extraction. To achieve reduction of hydroperoxides into hydroxides, 50 mM SnCl_2_ in methanol was added to the reaction mixture to the ratio water:methanol of 1:1 (v/v). After 10 minutes reduction, one volume of chloroform was added to obtain final ratio of water:methanol:chloroform of 1:1:1 (v/v/v), and the extraction of the reduced products was obtain as previously described.

### HPLC analysis of LiLOX oxidation products

Oxidized MGDG were separated on reversed phase high pressure liquid chromatography (RP-HPLC), using the column EC250/2 Nucleosil C_18_ (250 x 4 mm, 5 µm particle size, Macherey-Nagel, Düren, Germany) assembled in an Agilent 1100 HPLC system coupled to a diode array detector (Agilent, Waldbronn, Germany). Galactolipids were separated with solvent system A: acetonitrile:water:acetic acid 50:50:0.1 (v/v/v); B: acetonitrile:water:acetic acid 100:0:0.1 (v/v/v). After resolving the products in solvent A, the mixture was injected on the column with a flow rate of 0.2 mL/min, following a modified method from (Ibrahim et al., 2011). The following gradient was run for sample separation: From 0 to 10 min 0% solvent B. From 10 to 30 min, gradient from 0% to 100% of solvent B. From 30 min to 60 min, isocratic run. At min 60, post run to equilibrate the column in solvent A.

Transesterification was achieved with sodium methoxide in methanol and extracted with *n*-hexane. Oxidized methyl esters were separated on RP-HPLC, using the column EC250/2 Nucleosil C_18_. After transesterification, fatty acid methyl esters were dissolved in solvent system A: methanol:water:acetic acid 75:25:0.1 (v/v/v) and B: methanol:water:acetic acid 100:0:0.1 (v/v/v). The separation method was as follows: From 0 min to 5 min, isocratic run at 0% solvent B, with flow rate of 0.18 mL/min. From 5 min to 20 min, gradient from 0% to 100% solvent B and increased flow rate of 0.36 mL/min. From 20 min to 30 min, isocratic run. From 30 min to 35 min, gradient from 100% to 0% solvent B. At min 40, flow rate is back to 0.18 mL/min, post run until min 46.

Oxidized methyl esters were further analyzed on straight phase (SP) column Zorbax RX-SIL; (150 x 2.1 mm, 5 µm particle size, Agilent, Waldbronn, Germany) with flow rate of 0.2 mL/min in a solvent system containing *n*-hexane:2-propanol:trifluoroacetic acid 100:1:0.1 (v/v/v). Once the compounds of interest were collected, the stereoisomers were separated on chiral phase (CP) column CHIRALCEL OD-H (150 x 2.1 mm, Daicel Chiral Technologies, West Chester, PA, USA) with flow rate 0.1 mL/min. The solvent system consisted of *n*-hexane:2-propanol:trifluoroacetic acid 100:5:0.1 (v/v/v) for all HODE and HOTE. Absorbance was constantly recorded at wavelength 234 nm and 220 nm.

### *Ex vivo* assay

To identify natural substrates of *L. incisa* for the LiLOX activity a non-targeted *ex vivo* metabolome approach was performed as described recently (Feussner and Feussner, 2019; Ni and Feussner, 2022). A total metabolite extract of *L. incisa* was generated following a two-phase methyl-*tert*-butylether (MTBE) extraction modified from former publication (Matyash et al., 2008). Six parallel extractions of 20 mg lyophilized algae material each (normal growth conditions) were used. To three of the six extractions, 250 µg free fatty acid (FFA) 18:2 (n-6) and 250 µg FFA 20:4 (n-6) were added before starting the MTBE extraction. These extracts were used later in the enzymatic assay as positive control because both FFA are known substrates. Samples were dissolved in 0.5 mL 20 mM Bis-TRIS propane buffer, vortexed for 10 min and solubilized further by incubating in an ultrasonic water bath for 10 min. The dissolved extracts were centrifuged at 1500 x *g* for 10 min. The supernatants of all samples were split into two identical aliquots of 0.2 mL and transferred to reaction vials. 2 µg of LiLOX were added to one of the two aliquots. The second aliquot (without enzyme) was used as negative control. All reaction vials were incubated for 1 h at room temperature. The reaction was stopped by addition of acetonitrile. After 10 min of centrifugation at 1500 x *g*, the supernatant was directly used for measurement by non-targeted analysis.

For non-targeted analysis the samples were analyzed by ultra-performance liquid chromatography (UPLC, ACQUITY UPLC System, Waters Corporation, Milford, MA, USA) coupled to an electrospray ionization time-of-flight mass spectrometer (ESI-TOF-MS, LCT Premier, Waters Corporation, Milford, MA, USA). The UPLC was equipped with an ACQUITY UPLC HSS T3 column (1.0 x 100 mm, 1.8 µm particle size, Waters Corporation, Milford, MA, USA) and was used at 40°C with flow rate of 0.2 mL/min. The following gradient was run for sample separation: 0 – 0.5 min 40% B, 0.5 - 6 min 40% B to 100% B, 6 - 12 min 100% B, 12 - 12.1 min 100% B to 40% B, 12.1 - 15 min 40% B; (solvent system A: water:formic acid 100:0.1 (v/v); B: acetonitrile:formic acid 100:0.1 (v/v).

The TOF-MS analysis was recorded in the mass range of m/z 85 - 1200 for positive ESI and m/z 50 - 1200 for negative ESI mode over a runtime of 10 min using a capillary voltage of 2700 V (positive ESI) and 2500 V for negative ESI mode, respectively. The following source parameters were used: cone voltage, 30 V; desolvation temperature, 350°C; source temperature, 80°C. Nitrogen was used as cone and as desolvation gas at 30 l/h and 800 l/h, respectively. The mode of dynamic range enhancement was used for data acquisition. Data were recorded by MassLynx software (MassLynx V4.1, Waters Corporation, Milford, MA, USA) in centroid format. Mass analyses were adjusted by applying the reference spray compound leucine-enkephaline ([M+H]^+^ 556.2771 and the respective 2 x ^13^C isotopologue ([M+H]^+^ 558.2837) for positive ESI mode and [M-H]^-^ 554.2615 and as well as the respective ^13^C isotopologue ([M-H]^-^ 555.2648) for the negative ESI mode (Sigma-Aldrich, Deisenheim, Germany) at a concentration of 0.5 µg/mL in acetonitrile:water 50:50 (v/v) and a flow rate of 20 µL/min.

The raw data obtained by UPLC-TOF-MS analysis were subsequently used for data deconvolution (peak picking and peak alignment) by MarkerLynx Application Manager for MassLynx software (Waters Corporation, Milford, MA, USA) to generate data matrixes. For peak detection, the following parameters were used: retention time range, 0.30 - 10.00 min, mass range, 50 - 1200 Da, extracted ion chromatogram window 0.03 Da. Apex track peak parameters were set to automatic. Data deconvolution led to data matrixes of 2893 features for the positive ESI-mode and 1616 features for the negative ESI-mode, respectively.

### Data processing

Further data processing steps like ranking and filtering, adduct correction, merging data sets, data base search as well as clustering and visualization of the data was supported by the MarVis toolbox (Kaever et al., 2015). For ranking and filtering of the features an ANOVA test was applied to obtain a subset of 1002 (positive ESI-mode) respective 422 (negative ESI-mode) high quality features with a p-value < 10^-3^. Adduct correction was performed for the following adducts: [M+H]^+^, [M+Na]^+^, [M+NH_4_]^+^ (for positively charged ions); [M-H]^-^, [M+CH_2_O_2_-H]^-^, [M+CH_2_O_2_+Na-2H]^-^ (for negatively charged ions). Subsequently the data sets were combined and used for automated data base search as well as for clustering and visualization by means of by means of one-dimensional self-organizing maps (1D-SOMs). The number of three clusters was used to represent 1424 features. To identify tentative LiLOX substrates and products an in-house database was created, which includes the exact mass information of all MGDG, DGDG and PC species calculated for the acyl chains known to exist in the *L. incisa* lipid species and the derived hydroxides and hydroperoxides.

### MS/MS fragmentation analyses by UHPLC-ESI-QTOF-MS

For MS/MS fragmentation analyses LiLOX reaction products were reduced by SnCl_2_, acidified, separated by RP-HPLC and subsequently analyzed by UHPLC-ESI-QTOF-MS (Feussner et al., 2022). For UHPLC, an Agilent 1290 Infinity UHPLC system (Agilent, Waldbronn, Germany) equipped with an ACQUITY UPLC HSS T3 column (2.1 x 100 mm, 1.7 µm particle size, Waters Corporation, Milford, MA, USA) was used at 40°C with a flow rate of 1 ml/min. The UHPLC was coupled to an Agilent 6540 UH Accurate-Mass-Q-TOF mass spectrometer (Agilent, Waldbronn, Germany). Same solvent systems and gradients were used as described in 3.7.7. The QTOF-MS was in negative ESI-mode with a source suitable for Agilent Dual Jet Stream Technology (Agilent, Waldbronn, Germany). Source parameters were as follows: gas temperature 300°C, gas flow 8 L/min, nebulizer pressure 35 psi, sheath gas temperature 350°C, sheath gas flow 11 L/min, VCap 3.5 kV, nozzle voltage 100 V. Collision energies from 20 eV - 40 eV were used for collision induced dissociation. For data acquisition, the MS was operated with the software Mass Hunter Workstation Acquisition (Version: B.05.01, Agilent, Waldbronn, Germany). The Mass Hunter Qualitative Analysis software (Version: B.06.00, Agilent, Waldbronn, Germany) was used for data analysis.

### Lipidomic analysis by UPLC-nano ESI-MS/MS

Analysis of lipids by UPLC-nano ESI-MS/MS molecular species analysis was performed as previously described, with some modifications (Tarazona et al., 2015; Herrfurth et al., 2021). The analysis was started by UPLC using an ACQUITY UPLC^®^ I-class system (Waters Corporation, Milford, MA, USA) equipped with an ACQUITY UPLC^®^ HSS T3 column (100 mm × 1 mm, 1 μm; Waters Corporation, Milford, MA, USA). Aliquots of 2 μL were injected, the flow rate was 0.1 mL/min and 0.13 ml/min, respectively, and the separation temperature was 35°C. Solvent B was tetrahydrofuran:methanol:20 mM ammonium acetate (6:3:1; v/v/v) containing 0.1% (v/v) acetic acid; and solvent A was methanol/20mM ammonium acetate 3:7 (v/v) containing 0.1% (v/v) acetic acid. All lipids were separated with linear binary gradients following the same scheme: start conditions (65, 80, or 100% solvent B) held for 2 min, linear increase to 100% solvent B for 8 min, 100% solvent B held for 2 min and re-equilibration to start conditions in 4 min. The start conditions were 80% solvent B for diacylglycerols (DAG), 100% solvent B for triacylglycerols (TAG) and 65% solvent B for all remaining lipid classes. For TAG, the isocratic chromatographic runs were finished after 8 min.

Chip-based nanoelectrospray ionization was achieved with a TriVersa Nanomate (Advion, Ithaca, NY, USA) in both positive and negative ion mode with 5 μm internal diameter nozzles. The ion source was controlled with the Advion ChipSoft Manager software. By using a post-column splitter 255 nL/min of the eluent were directed to the nanoESI chip and ionization voltage was set to either 1.4 kV (TAG) or 1.23 kV (all remaining lipid classes). Molecular lipid species were detected with a 6500 QTRAP tandem mass spectrometer (AB Sciex, Framingham, MA, USA) in multiple reaction monitoring mode. All theoretically possible molecular lipid species were calculated and measured by UPLC-nano ESI-MS/MS-based method as described above on the basis of the known fatty acid profile of *L. incisa* consisting of 14:0, 16:0, 16:1, 16:2, 16:3, 18:0, 18:1, 18:2, 18:3, 20:0, 20:1, 20:2, 20:3, 20:4 and 20:5 (Bigogno et al., 2002). The oxidized fatty acid species containing one oxygen as well as two oxygens of 16:2, 16:3, 18:2 and 18:3 were additionally taken into account for calculation of the molecular species of the glycerophospholipids and glyceroglycolipids. Target precursor ions were [M+NH_4_]^+^ for DAG and TAG; [M-H]^-^ for PE, PG, PS and SQDG; and [M-H+CH_3_CO_2_H]^-^ for PC, MGDG, DGDG. For diacyl and triacyl lipids, target selected reaction monitoring transitions were diagnostic for the acyl chain composition of the molecular species, either by a fatty acid-associated neutral loss in the positive mode (DAG and TAG) or by formation of fatty acyl-related fragments in the negative ion mode (glycerophospholipids and glyceroglycolipids). Dwell time was 5 ms and MS parameters were optimized to maximize detector response. The integration workflow made use of the Analyst IntelliQuan (MQII) peak-finding algorithm.

### Phytohormone measurements

Phytohormones were extracted with MTBE, reversed phase-separated using an ACQUITY UPLC system (Waters Corporation, Milford, MA, USA) and analysed by nanoESI (TriVersa Nanomate; Advion BioSciences, Ithaca, NY, USA) coupled with an AB Sciex 4000 QTRAP tandem mass spectrometer (AB Sciex, Framingham, MA, USA) employed in scheduled multiple reaction monitoring mode according to Herrfurth and Feussner, (2020). The reversed phase separation of constituents was achieved by UPLC using an ACQUITY UPLC HSS T3 column (100 mm x 1 mm, 1.8 μm; Waters Corporation, Milford, MA, USA). Solvent A and B were water and acetonitrile:water 90:10 (v/v), respectively, both containing 0.3 mmol/l NH_4_HCOO (adjusted to pH 3.5 with formic acid). Mass transitions were as follows: 209/59 [declustering potential (DP) -30 V, entrance potential (EP) -4.5 V, collision energy (CE) -24 V] for JA, 214/62 (DP -35 V, EP -8.5 V, CE -24 V) for D_5_-JA, 291/165 (DP -50 V, EP -5 V, CE -26 V) for OPDA, 296/170 (DP -65 V, EP -4 V, CE -28 V) for D_5_-OPDA, 322/130 (DP -45 V, EP -5 V, CE -28 V) for JA-Ile and 325/133 (DP -65 V, EP -4 V, CE -30 V) for D_3_-JA-Leu.

### Microscopy

The fluorescent proteins enhanced yellow fluorescent protein (eYFP) and enhanced cyan fluorescent protein (eCFP) were used to tag the proteins of interest, allowing them to be visualized by fluorescence microscopy. Two vectors pUC18-35S::LiLOX-YFP and pCAT-35S::ACP-CFP (Fulda et al., 2002) were used for transformation. 10 mg of gold particles (1 µm diameter, from Bio-Rad Laboratories GmbH, München, Germany) were washed with ethanol and rinsed in sterile distilled water. 0.2 mg of the particles were mixed with 8 µg of highly concentrated vector, 55 µL final volume. After vigorous agitation, 50 µL of CaCl_2_ (2.5 M) and 20 µL of spermidine (0.1 M) were added to precipitate the DNA. After thorough mixing and washing steps in ethanol, the freshly coated particles were resuspended in 60 µL ethanol. Particles were shot at freshly cut onion (*Allium cepa*) slices using a PDS1000/He Biolistic Particle Delivery System (Bio-Rad Laboratories GmbH, München, Germany). For each bombardment, 20 µL of particle suspension as well as rupture discs (max pressure: 1350 pounds per square inch (psi)) were used in vacuum (28 inches of mercury). Directly after bombardment onion slices were placed in a petri dish with wet tissues, and kept in the dark overnight at room temperature. The next morning, single layers of epithelial cells were collected using forceps, and placed on a glass slide for analysis.

### Growth conditions and transformation of *A. thaliana*

*A. thaliana* plants were cultivated on soil obtained from (Frühstorfer Erde Typ T fein, Hawita Gruppe GmbH, Vechta, Germany). Wildtype *A. thaliana* accession Col-0 and T-DNA insertion mutants were obtained from the Nottingham Arabidopsis Stock Centre (NASC). Line SALK_062064 (*LOX3*, At1g17420), line SALK_071732 (*LOX4*, At1g72520), line SALK_08365 (*LOX6*, At1g67560) and line *lox2-1* (*LOX2*, At3g45140) (Glauser et al., 2009) were crossed to generate a quadruple knockout without any active linoleate 13-LOX (*13S-lox* KO). *A. thaliana* plants were cultivated in a climate chamber (York Industriekälte GmbH & Co. KG, Hamburg, Germany) and transferred to the green house after the onset of flowering. Plants were cultivated under long day conditions (16 hours of light for 8 hours of dark) for propagation, transformation and selection of transgenic plants. For wounding experiments plants were cultivated under short day conditions (8 hours of light and 16 hours of dark). *13S-lox* KO plants are deficient in JA biosynthesis resulting in male sterility. Therefore, flowers of the quadruple KO were daily sprayed with 0.01% (v/v) methyl jasmonate in a solution containing 0.1% (v/v) Tween 20 to obtain seeds.

*Agrobacterium tumefaciens* (*A. tumefaciens*) strain EHA105 was used for *A. thaliana* transformation. Transformation of *A. tumefaciens* was performed with 3 µg plasmid DNA (pCAMBIA33-35S::LiLOX) following a modified version of (Höfgen and Willmitzer, 1988). *A. thaliana* plants were then transformed by flower dipping. Plants were cultivated in the green house, and the dipping procedure was repeated after 1 week. T1-seeds were harvested after 2 to 4 weeks. Transgenic plants were selected *via* herbidicide treatment (BASTA, Bayer. Leverkusen, Germany).

## Results

### Endogenous transcripts of LiLOX accumulate during *L. incisa* nitrogen starvation

**Figure 1:**
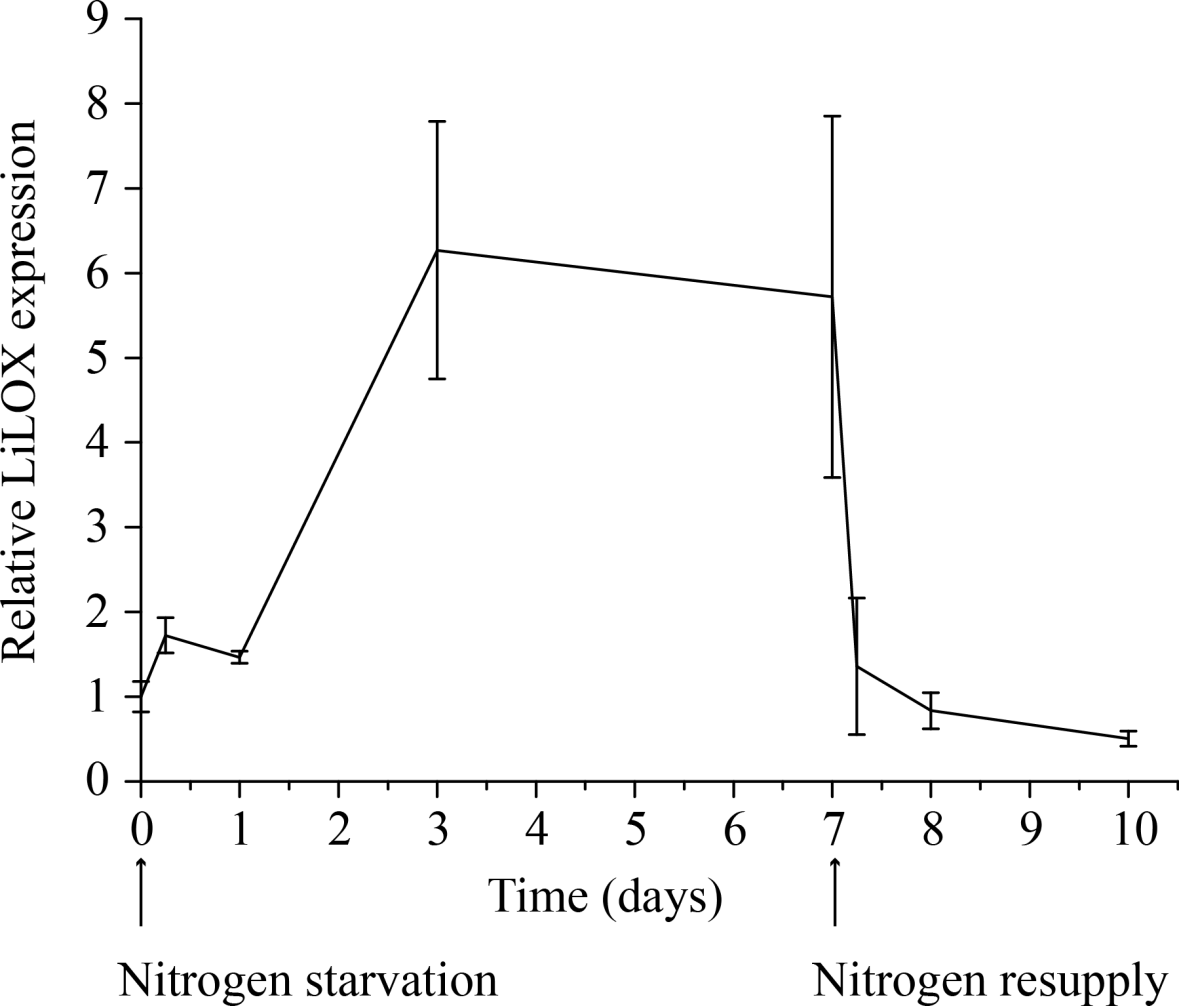
Transcript level of LiLOX during nitrogen starvation. Cultures of *L. incisa* were grown in full BG11 media. The cells were then split in six different cultures, three with full BG11 medium and three without any nitrogen source. Cells were collected at different time points, and RT-qPCR was performed to measure the transcript level of LiLOX and ribosomal protein S21 as control. The graph shows the difference of LiLOX expression after nitrogen starvation compared to growth in full media, normalized to the reference gene. Error bars represent the standard deviation of the three samples grown in parallel.

We recently identified a linoleate 13-lipoxygenase (LiLOX) and we reasoned that it may influence the accumulation of PUFAs in TAGs upon nitrogen starvation (Djian et al., 2019). In order to address this hypothesis, we analyzed its transcript induction in *L. incisa* cells which were grown in parallel, either with or without nitrogen supply. Transcripts of LiLOX were measured by real-time quantitative PCR (RT-qPCR). Indeed, results show a clear accumulation of LiLOX transcripts (7 fold) during nitrogen starvation (**Figure 1**). LiLOX transcripts remained high for as long as starvation was tested and dropped to baseline after transfer in nitrogen-containing media.

### LiLOX has plastidic sub-cellular localization

**Figure 2:**
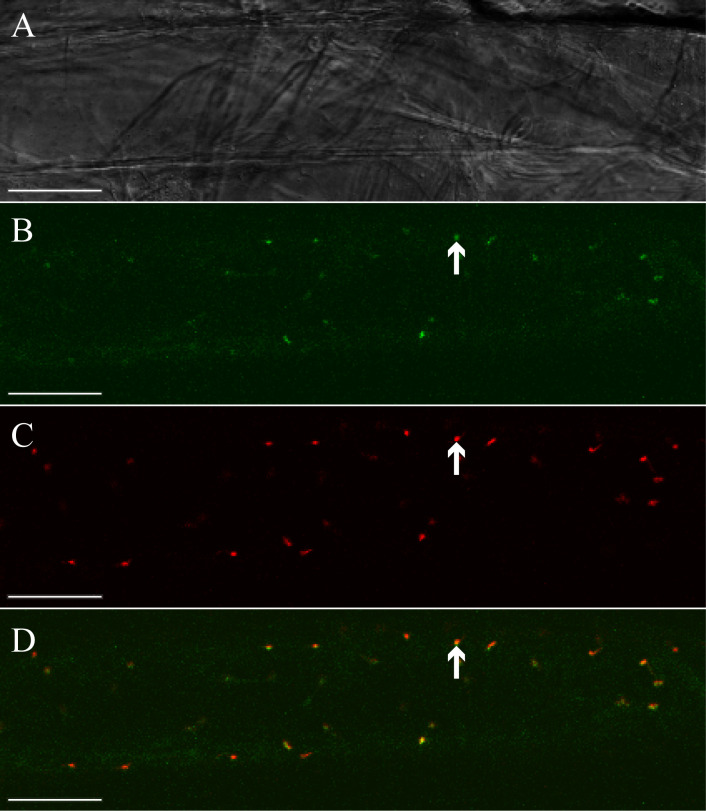
Subcellular localization of LiLOX yellow fluorescence fusion protein (LiLOX-eYFP). Confocal microscopy of epithelial onion cell co-transformed with LiLOX-eYFP and acyl carrier protein fused to cyan fluorescence protein (ACP-eCFP). **(A)** Bright field. **(B)** LiLOX-eYFP. **(C)** ACP-CFP. **(D)** Merged signals of LiLOX-eYFP and ACP-eCFP. All 10 cells analyzed from a single experiment showed comparable results. White Bar: 100 μm.

Based on its sequence identity with plastidic LOXs from flowering plants, we next analyzed whether LiLOX has a plastidic localization (Djian et al., 2019). To show its subcellular localization, LiLOX was fused to the yellow fluorescent protein (YFP) at its C-terminus and expressed transiently in onion epidermal cells. The acyl carrier protein (ACP) fused to the cyan fluorescent protein (CFP) at its C-terminus was used for co-localization. The analysis by fluorescent microscopy confirmed that LiLOX harbors a plastidial signal peptide, as it co-localizes together with ACP to the stroma of plastids of onion epidermal cells (**Figure 2**).

### LiLOX can oxidize complex lipids

**Figure 3:**
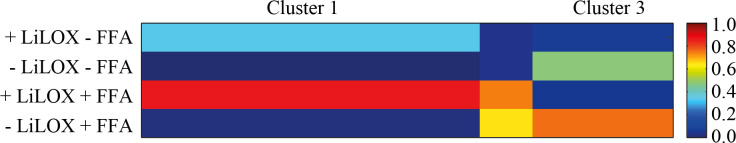
*Ex vivo* assay of LiLOX. After extraction of the total metabolites from *L. incisa*, the mixture was solubilized in bis-tris propane buffer pH 7.5, and split in four samples named A, B, C and D. Purified LiLOX was added to the samples A and C. Pure free PUFAs (FFA) were added to samples C and D as internal standards. All samples were analyzed following a non-targeted method, by LC-MS, and the data were analyzed by automatic clustering using the software MarVis. The figure represents the One-dimensional Self-Organizing Map (1D-SOM) revealing putative substrates and putative products with and without LiLOX reaction. The heat map colors represent average intensity values according to the color map on the right-hand side. The width of each cluster is proportional to the number of features assigned to this cluster.

Next we aimed to analyze the endogenous substrates of LiLOX by *ex-vivo* metabolomics (Feussner and Feussner, 2020). Therefore, a metabolite extract of *L. incisa* cells was solubilized in a buffer appropriate for LiLOX activity. The extract was split in 4 identical samples. LiLOX was expressed heterologously in *E. coli*, purified as described previously and 2 µg of LiLOX was added to two of the samples (Djian et al., 2019). A mixture of 18:2 (n-6) and 20:4 (n-6) was added additionally as internal positive control to two of the samples. After an hour, the reaction was stopped by adding acetonitrile and analyzed by a non-targeted metabolome fingerprinting workflow with positive and negative ionization (Feussner et al., 2022). The software MarVis was used for data preprocessing such as ranking, filtering, adduct correction and data merging (Kaever et al., 2015). Finally, 1424 features with pVal below 10^-3^ were selected for clustering by one-dimensional self-organizing maps (1D-SOMs, **Figure 3**). Among all high-quality features, oxidized MGDG as well as oxidized PC species were highly enriched in the fraction with active LiLOX (Cluster 1), and absent in the fractions without enzyme (Cluster 3) suggesting that LiLOX can oxidize plastidic membrane lipids.

### LiLOX oxidizes the plastidic lipid MGDG from *L. incisa* with high preference

**Figure 4:**
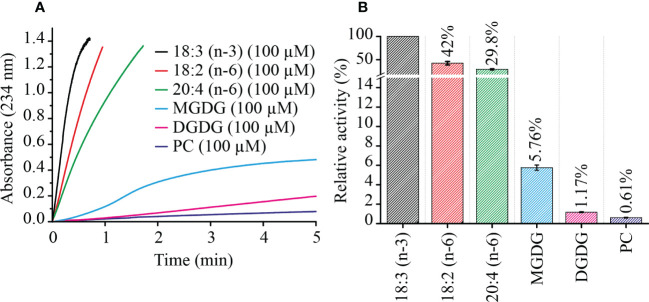
Kinetics of LiLOX with PUFAs and complex lipids extracted from *L. incisa*. **(A)** velocity of LiLOX reaction as measured spectrophotometrically at a wavelength of 234 nm. **(B)** Initial velocities of LiLOX with different substrates, relative to 18:3 (n-3). Error bars represent the standard deviation of three reactions from the same enzyme preparation.

In order to confirm this finding from the non-targeted *ex-vivo* approach, MGDG, DGDG and PC were extracted from *L. incisa* cells, by total lipid extraction following separation of these lipid classes on TLC plates. LOX reactions were performed in Bis-TRIS propane buffer pH 7.5, with 100 µM of each complex lipid separately and 0.1% of sodium deoxycholate to ensure solubility. Velocity of LOX reactions were measured by a photometric assay (234 nm, the absorbance of the formed conjugated diene system). For comparison, initial LOX velocity was also measured with the three most abundant PUFAs of *L. incisa*: 18:2 (n-6), 18:3 (n-3), and 20:4 (n-6) (Lang et al., 2011). Confirming previous results LiLOX was found to have maximum velocity with 18:3 (n-3), followed by 18:2 (n-6) and finally 20:4 (n-6) (Djian et al., 2019). In case of complex lipids, all attempts to measure the kinetic parameters in the presence of detergents failed since the measured data fitted neither the Michaelis-Menten nor the Hill equation. Therefore, reaction rates were measured for all substrates at the same concentration. LiLOX had a clear preference towards MGDG as its initial velocity was found to be 10 times faster than with PC (**Figure 4**).

### LiLOX oxidizes MGDG, DGDG and PC at acyl carbon n-6 with strict *S* configuration

**Figure 5:**
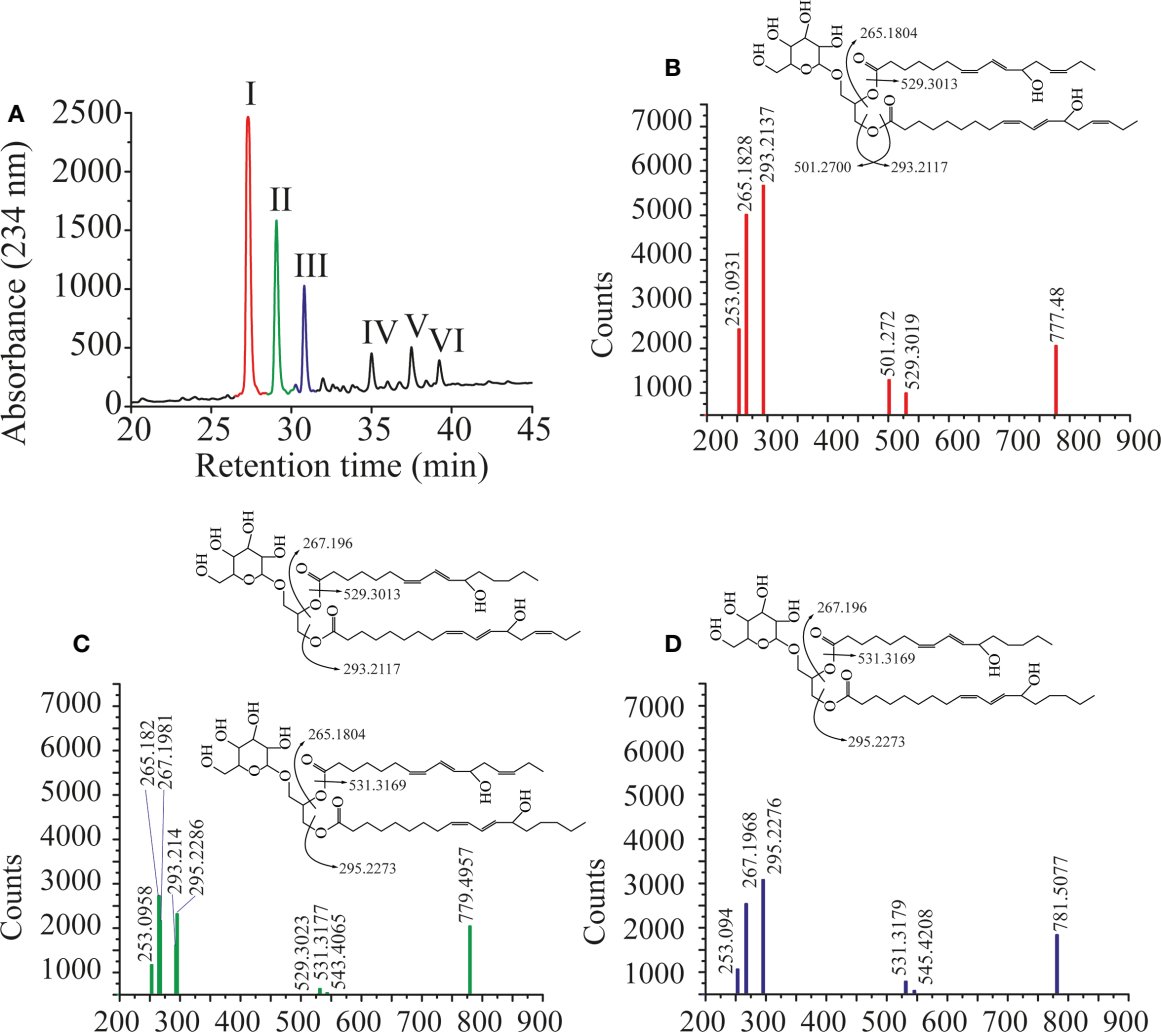
Oxidation products of LiLOX with MGDG isolated from *L. incisa*. **(A)** RP-HPLC of all oxidation products after chemical reduction. Six peaks were identified, named I, II, III, IV, V, and VI. B-D. Fragmentation pattern of the purified features I, II and III obtained by LC-MS/MS. The position of the hydroxyl groups on the chemical structures shown are tentatively assigned. **(B)** Peak I. **(C)** Peak II. **(D)** Peak III. Data shown represent a single experiment.

**Figure 6:**
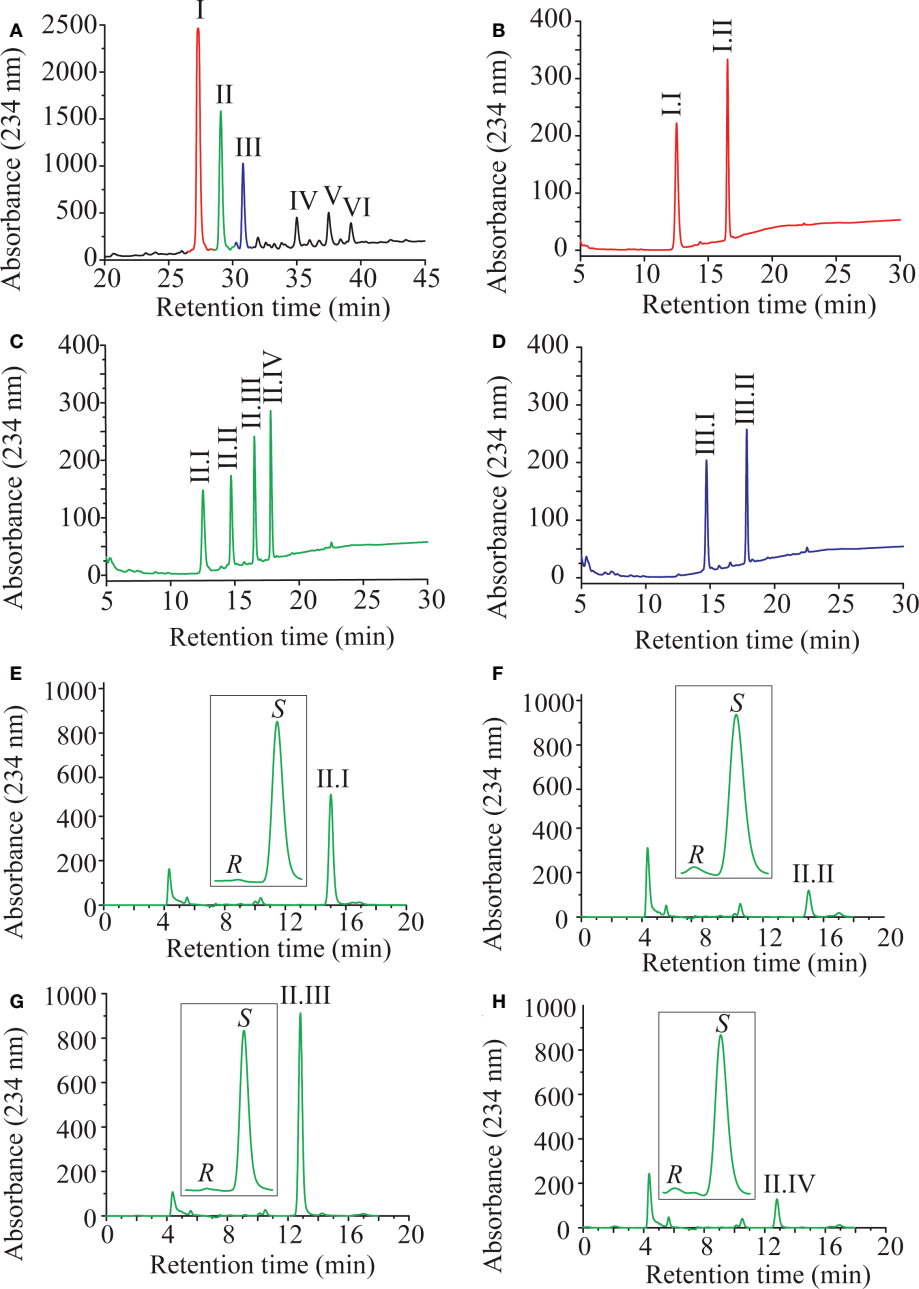
Sequential HPLC analysis of oxidation products of LiLOX with MGDG isolated from *L. incisa*. **(A)** Purification of the oxidized MGDG products by RP HPLC (same Figure as in 5A). **(B-D)** After transmethylation of the acyl chains from the peaks I; II and III, the mixtures of oxidized fatty acid methyl esters were purified one more time by RP HPLC. **(B)** Peak I. **(C)** Peak II. **(D)** Peak III. **(E-H)** Sequential analysis of the oxidized fatty acid methyl esters from the peak II by SP HPLC and CP HPLC. **(E)** Peak II.I: 11*S*-HHTE. **(F)** Peak II.II: 11*S*-HHDE. **(G)** Peak II.III: 13*S*-HOTE. **(H)** Peak II.IV: 13*S*-HODE. The identity of the methyl-esters was confirmed by authentic standards. RP-HPLC and SP-HPLC chromatograms are representative of at least three measurements with independent enzyme preparations. CP-HPLC chromatograms are representative of single measurements.

Previously LiLOX was identified as linoleate 13- and arachidonate 15-LOX when free fatty acids were used for the analysis (Djian et al., 2019). In light of the identification of membrane lipids as substrates, we wondered whether this positional specificity was the same with complex lipids. Since LiLOX showed highest activity with MGDG, its oxidation products were analyzed first by RP-HPLC. It revealed six separate peaks with an absorption maximum at 234 nm (**Figure 5A**). They were numbered according to their retention time (RT; I, II, III, IV, V and VI respectively) and collected. The first three major ones were further analyzed by LC-MS/MS (**Figures 5B-D**). Their masses corresponded to the substrates 34:6-MGDG, 34:5-MGDG and 34:4-MGDG respectively with two additional oxygens. The masses of peaks IV, V and VI, corresponded to the same substrates with only one additional oxygen (**Supplementary Figure 1**). This result highly suggests that LiLOX can oxidize MGDG species on both acyl chains, in a two-step reaction. Fragmentation by MS/MS confirmed this result, as the fragment masses of peak I were equivalent to a HOTE- and a hydroxy hexadecatrienoic acid (HHTE)-residue. The mass fragments of peak II were equivalent to a HOTE-, a HODE-, a HHTE- and a hydroxy hexadecadienoic acid (HHDE)-residue, revealing that peak II was a mixture of two MGDG isomers (HOTE/HHDE-MGDG and HODE/HHTE-MGDG respectively). The mass fragments of peak III were equivalent to a HODE- and a HHDE-residue. On the other hand, fragmentations of peaks IV, V and VI revealed oxidations on the 18-carbon acyl chains, but were never observed with oxidation on the 16-carbon acyl chains (**Supplementary Figure 1**), highly suggesting that the two steps of the LiLOX lipid oxidation reaction starts on the 18-carbon moiety and oxidizes the 16-carbon acyl chain afterwards. To determine the absolute configuration of the acyl chains, the peaks I, II and III were transesterified and sequentially analyzed by RP-HPLC, straight phase (SP)-HPLC and chiral phase (CP)-HPLC (**Figure 6**). All fatty acid methyl esters were found to be oxidized on the carbon n-6 with strict *S* configuration.

Similar to the MGDG fraction, DGDG and PC fractions oxidized by LiLOX were analyzed by LC-MS, revealing that most DGDG and some PC molecular species were oxidized on both acyl chains (data not shown). Yet contrary to the MGDG fraction, not all DGDG and PC species were found to be oxidized by LiLOX, probably due to the unsaturation levels of the DGDG and PC fractions in *L. incisa*. The mixture of DGDG and PC were trans-esterified and sequentially analyzed by RP-, SP-, and CP-HPLC (**Supplementary Figures 2** and **3**). Similar to MGDG, all fatty acid methyl esters were found to be oxidized on the carbon n-6 with strict *S* configuration (**Table 1**).

**Table 1:** All oxidation products of MGDG, DGDG and PC isolated from *L. incisa* after LiLOX reaction.

	HHTE	HHDE	HOTE	HODE	HETE
MGDG: 34:4;2	0%	7.9%	0%	7.1%	0%
		7-HHDE: 18.6%		9-HODE: 3.9%	
		11-HHDE: 81.4% (92.2% *S*)		13-HODE: 96.1% (100% *S*)	
MGDG: 34:5;2	8.1%	6.5%	7.2% α	7.7%	0%
	7-HHTE: 0%	7-HHDE: 0%	9-HOTE: 0%	9-HODE: 0%	
	10-HHTE: 0%		12-HOTE: 0%		
	11-HHTE: 97.6% (99% *S*)	11-HHDE: 83.1% (98.1% *S*)	13-HOTE: 97.0% (98% *S*)	13-HODE: 87.8% (96.6% *S*)	
	14-HHTE: 0%		16-HOTE: 1.5%		
	All trans: 2.4%	All trans: 16.9%	All trans: 1.4%	All trans: 12.2%	
MGDG: 34:6;2	28.5%	0%	26.9% α	0%	0%
	7-HHTE: 0%		9-HOTE: 0%		
	10-HHTE: 0%		12-HOTE: 0%		
	11-HHTE: 99% (87.9% *S*)		13-HOTE: 87.1% (97.4% *S*)		
	14-HHTE: 0%		16-HOTE: 12.9%		
DGDG mixture	9.10%	12.90%	28.40% α	42.20%	7.40%
		All trans: 7.6%	all trans: 0.7%	All trans: 1.2%	5-HETE: 0%
		7-HHDE: 0%		9-HODE: 2.3%	8-HETE: 7.2%
	7-HHTE:		9-HOTE: 0%		10-HETE: 0%
	10-HHTE:	11-HHDE: 91.6% (96.8% *S*)	12-HOTE: 0%	13-HODE: 96.5% (98.9% *S*)	11-HETE: 0%
	11-HHTE: 100% (87.6% *S*)		13-HOTE: 99.3% (97.1% *S*)		12-HETE: 0%
	14-HHTE:		16-HOTE: 0%		15-HETE: 92.7% (87.1% *S*)
PC mixture	0%	0%	6.0% α/7.3% γ	59.40%	27.50%
				9-HODE: 1.9%	5-HETE: 0%
					8-HETE: 0%
			9-HOTE: 11.2%		10-HETE: 0%
			12-HOTE: 8.4%	13-HODE: 96.8% (87% *S*)	11-HETE: 2.3%
			13-HOTE: 78.2% (100% *S*)		12-HETE: 0%
			16-HOTE: 0%		15-HETE: 96.0% (87.3% *S*)

All reactions were performed in 20 mM Bis-TRIS propane buffer with 0.1% w/v sodium deoxycholate. Products were reduced by SnCl_2_, transesterified and analyzed sequentially by RP-HPLC, SP-HPLC and CP-HPLC. Oxidized acyl chains mixtures from a given lipid class analyzed by RP-HPLC are represented in red. In blue are the major regioisomers for each oxidized acyl chain. The identity of the methyl-esters was confirmed by authentic standards. Chiral configuration was measured for all major regioisomers, shown in brackets. Stereochemistry for HHTE-Me and HHDE-Me was tentatively assigned, due to the absence of corresponding standards. HHTE, hydroxy hexadecatrienoic acid, HETE, hydroxy eicosatetraenoic acid; HODE, hydroxy octadecadienoic acid; HOTE, hydroxy octadecatrienoic acid; nd, not determined.

Since these data revealed that the MGDG and PC fractions extracted from *L. incisa* had unequal unsaturation levels (**Supplementary Table 1**), now the purified molecular species 36:6-MGDG (18:3 (n-3)/18:3 (n-3)-MGDG), 36:6-PC (18:3 (n-3)/18:3 (n-3)-PC) as well as the PUFA 18:3 (n-3) were used as substrates. As expected, the velocity of LiLOX towards 36:6-PC was highly increased (10 fold) compared to the PC fractions extracted from *L. incisa*, assuredly due to the strong difference in unsaturation levels from the two substrates (**Supplementary Figure 4**).

### Mutations on Q691 affect the enzyme velocity for specific substrates

**Figure 7:**
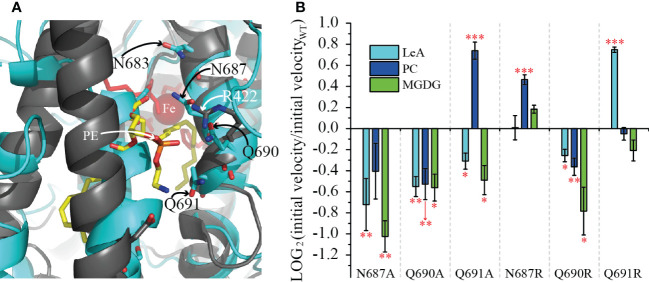
Effect of the mutations of the residues N687, Q690 and Q691 on the reaction velocity of LiLOX with different substrates. **(A)** Superimposition of the modeled structure of LiLOX (black labels) and PaLOX (white labels, PDB accession number: 5IR5). Shown is the entrance of the substrate channel. The modeled structure of LiLOX is depicted as cyan cartoon and sticks. PaLOX is depicted as black cartoon and sticks, the substrate PE in yellow sticks and the iron as red sphere. **(B)** velocity of LOX reaction from mutant enzyme, relative to the WT enzyme (**Supplementary Figure 4**). LeA: 18:3 (n-3). PC: 18:3 (n-3)/18:3 (n-3)-PC. MGDG: 18:3 (n-3)/18:3 (n-3)-MGDG. Each bar represents the average velocity of three measurements. Each measurement was performed with an independent enzyme preparation. Statistical analysis was carried out using Student’s t-test. Asterisks indicate different significance levels at ***, P < 0.001, **, P < 0.01 and *, P < 0.1 compared with the WT.

Next, we aimed to analyze the structural determinants of the substrate specificity of LiLOX against complex lipids. Therefore, we used the structural model of the enzyme described recently (Djian et al., 2019). Yet, regardless of the substrate unsaturation level, three amino acids present at the entrance of the substrate channel of LiLOX were suspected to play a role in the substrate recognition: N687; Q690; Q691 (**Figure 7A**). The side chains of these three amino acids harbor an amide group, able to form hydrogen bonds with the alcohol groups from the galactosyl moiety from MGDG. All three amino acids were independently mutated into alanine and arginine. As results, all three alanine exchanges, N687A; Q690A and Q691A decreased significantly the velocity of LiLOX towards 36:6-MGDG, supporting the hypothesis that these three residues have a role in stabilizing MGDG during catalysis (**Figure 7B**). On the other hand, Q691A and N687R increased significantly the velocity of LiLOX towards 36:6-PC, and the mutation Q691R increased significantly the velocity of LiLOX towards 18:3 (n-3). Together, Q691 seemed to be particularly important, since its mutation into alanine increased the velocity specifically for PC by 167% and its mutation into arginine increased the velocity specifically for 18:3 (n-3) by 168%.

### LiLOX seems to perform an additional reaction with MGDG

When the oxidation of MGDG by LiLOX was followed spectrophotometrically for longer time periods, it turned out that the reaction can be divided in two phases. First, an increase of the absorbance at 234 nm can be observed which indicates the formation the conjugated double bond system. Second, the absorbance at 234 nm decreased again after 10 minutes (**Supplementary Figures 5A, B**). This observation suggests that the conjugated double bond system must be transformed into a new functional group, possibly by a second reaction with LiLOX. Moreover, this phenomenon was never observed with free 18:3 (n-3) (**Supplementary Figures 5C, D**), suggesting a specificity of this second reaction for complex lipids.

To confirm this second step, the reaction was stopped at different time points by dilution in pure ethanol (1:1 v/v). The products were then analyzed by LC-MS without any further processing. As expected, the share of MGDG substrates were quickly metabolized after 10 minutes. Products with 2 additional oxygens and 4 additional oxygens were formed sequentially, confirming the oxidation of the two acyl chains in two steps. After 10 minutes of reaction, 36:6-MGDG substrates as well as 36:6;2-MGDG and 36:6;4-MGDG products decreased in the reaction mixture, yet no additional products were detected (**Supplementary Figure 6**). The fact that MGDG substrates were detected after LiLOX reaction with 6 additional oxygen atoms, suggests that LiLOX might perform an additional reaction adding 2 oxygens after both acyl chains have been oxidized. A reaction of purified 13*S*-HOTE/11*S*-HHTE-MGDG with LiLOX, recorded by HPLC and with an oxygen electrode, could confirm an addition of dioxygen. Nevertheless, even these compounds do not seem to be the final LiLOX products, as they decrease between 30 minutes and 60 minutes. Since it was described before for a LOX from soybean and two LOXs from *Physcomitrium patens* to possess fatty acid hydroperoxide lyase activity all recorded data sets were first analyzed for the occurrence of the potential fragments of the corresponding lipid hydroperoxides and in a second step in a completely untargeted way for new products (Salch et al., 1995; Senger et al., 2005). However, by neither of the two strategies we were able to identify the final endogenous MGDG-derived product of LiLOX.

### Nitrogen starvation led to reduction of plastid size as well as accumulation of lipid droplets

Since LiLOX transcript was shown to increase upon nitrogen starvation (**Figure 1**), this experimental setup was used to profile all major lipid classes that may serve as LiLOX substrates. Cells of *L. incisa* were grown in six liquid cultures; three of them in full BG11 media, and three in absence of nitrogen source (BG11N-). Samples were collected at different time points, and lipidomic analysis was performed by UHPLC-ESI-QTOF-MS. The results showed an overall decrease of plastidic lipids, and an accumulation of TAG in the cells grown in absence of nitrogen (**Supplementary Figure 7** and **Supplementary Table 1**). However, no LOX product was detected under these conditions. The drastic lipid remodeling within 5 days of nitrogen starvation was accompanied by the formation of lipid droplets shown by BODY P staining and a reduction of the size of the plastid (chlorophyll fluorescence) by fluorescent microscopy (**Supplementary Figure 8**) confirming a previous publication (Merzlyak et al., 2007).

### LiLOX complements the jasmonate levels in an *Arabidopsis thaliana* 13-LOX KO

**Figure 8:**
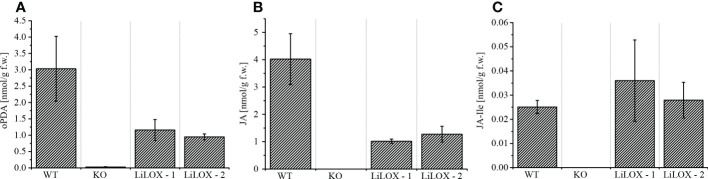
JA pathway metabolites in *A. thaliana 13-lox* KO. WT: *A. thaliana* Col-0. KO: *A. thaliana* Col-0 from which all *13-LOX* (*AtLOX2*, *AtLOX3*, *AtLOX4* and *AtLOX6*) were knocked out. LiLOX-1 and LiLOX-2 are two lines of *A. thaliana 13S-lox* KO, complemented with LiLOX under the 35S promotor. Two hours after wounding leaves were harvested and extracted. Phytohormone levels were measured by LC-MS/MS. Error bars represent the standard deviation of three measurements. All three measurements were performed from three different plants grown in parallel. **(A)** oPDA. **(B)** JA. **(C)** JA-Ile.

To detect LiLOX activity endogenously, LiLOX was used to complement the jasmonic acid (JA) pathway in an *A. thaliana* mutant in which all *13-LOX* (*AtLOX2*, *AtLOX3*, *AtLOX4* and *AtLOX6*) were knocked out. Since these four 13-LOX are the only enzymes that can synthesize the first precursor of the JA synthesis in *A. thaliana*, the quadruple *13-lox* KO is JA free and therefore male sterile (Ishiguro et al., 2001). Phytohormone analysis 2 hours after wounding confirmed the JA depletion in the quadruple *13-lox* KO strain (**Figure 8**). Transformation of the quadruple *13-lox* KO with 35S::LiLOX successfully rescued oPDA, JA and JA-Ile in the leaves 2 hours after wounding. RT-qPCR confirmed expression of LiLOX transcripts in *A. thaliana* leaves (data not shown). Yet *A. thaliana* mutant complemented with 35S::LiLOX did not recover male fertility, impaired by the quadruple *13-lox* KO (data not shown), most likely because the expression of LiLOX was in the flowers bellow the necessary threshold.

## Discussion

Knowledge on lipid oxidation processes in microalgae is scarce and the major source of enzymatic lipid oxidation in land plants are LOXs (Andreou et al., 2009; Wasternack and Feussner, 2018). Therefore, the aim was to close this gap by analyzing the recently identified LOX of the green alga *L. incisa* in more detail (Djian et al., 2019). This previous study showed that LiLOX and plastidic LOXs from vascular plants share a common ancestor, based on their sequence homology. Besides this sequence homology, the study demonstrated that LiLOX has a similar pH optimum, positional and stereo specificity as all so far characterized plastidic LOXs (Wasternack and Feussner, 2018). Here, we provide further evidence by showing that LiLOX is indeed targeted to the stroma of plastids of onion epidermal cells (**Figure 2**). Within the plastid, all LOXs are found so far to be located in the stroma (Blée and Joyard, 1996; Liavonchanka and Feussner, 2006). The pH optimum of LiLOX (pH 7.5) would be in agreement with a location inside the stroma, since its pH is either around 7.1 during the night or around 7.9 during the day when photosynthesis takes place (Robinson, 1985; Tikhonov, 2012). Now we provide additional evidence by showing that LiLOX complemented an *A. thaliana* quadruple plastidic *13-lox* KO by rescuing JA biosynthesis in its wounded leaves (**Figure 8**). All these findings are in agreement with the hypothesis that LiLOX and plastidic LOXs in vascular plants share a common ancestor and most likely a conserved function.

A major finding of this report is that LiLOX was *in vitro* not only able to oxidize free PUFAs, but in addition it oxygenates acyl chains esterified to PC and to the plastidic lipids MGDG and DGDG (**Figures 3**-**6**). While it is widely accepted that certain LOXs metabolize phospholipids under physiological conditions (Brash, 1999), results obtained in this study provide evidence that LiLOX accepts in addition glycolipids such as MGDG and DGDG. Chloroplasts have been described to harbor a number of different lipid classes and the most abundant ones are galactolipids, in particular MGDG and DGDG (Hölzl and Dörmann, 2019). Other plastidic lipids such as sulfoquinovosyldiacylglycerol and phosphatidylglycerol are highly saturated lipids in *L. incisa* and are therefore very likely no natural substrate of LiLOX (Bigogno et al., 2002). However, all acyl chains of MGDG were found to harbor at least one pentadiene system and only two species of DGDG out of 10 have acyl chains harboring fewer than 4 double bonds and therefore the two galactolipid classes MGDG and DGDG represent indeed very good natural substrates of LiLOX (**Supplementary Table 1**). Over the years only a few plant LOXs were found to accept complex lipids as substrates such as phospholipids, MGDG, DGDG and TAG (Kondo et al., 1993; Feussner et al., 1995; Holtman et al., 1997; Feussner et al., 1998; Ibrahim et al., 2011; Nakashima et al., 2011). However, it should be pointed out that except for LOX2 from *A. thaliana*, experimental evidence showing specifically that a plastidic LOX metabolizes glycolipids is still missing (Glauser et al., 2009; Zoeller et al., 2012; Mochizuki et al., 2016; Mochizuki and Matsui, 2018). This study provides experimental evidence that a purified recombinant plastidic LOX oxidizes MGDG and DGDG *in vitro* (**Figures 3**-**6**). After product analysis, all molecular species of MGDG purified from *L. incisa* were found to be oxidized by LiLOX on both acyl chains (**Figure 5**). A similar result was observed for 8 out of 10 DGDG molecular species, whereas only 4 PC molecular species out of 9 were accepted as substrate by LiLOX (**Supplementary Figures 2** and **3**). Even if part of these results can be explained by the saturation level of these lipid classes, it cannot be the only explanation. Indeed, the PC molecules 34:2, 36:3, and 38:5 were not found to be oxidized at all although these species in *L. incisa* were found to have at least one pentadiene system that can serve as substrate (**Supplementary Table 1**).

In order to compare the activity of recombinant LiLOX towards complex lipids, the oxidation rates were followed with MGDG, DGDG and PC fractions purified from *L. incisa* and reaction rates were measured for all substrates at the same concentration (**Figure 4**). Under these conditions, MGDG was found to be oxidized 4.9 times faster than DGDG, and 9.4 times faster than PC *in vitro*. One factor that can explain this phenomenon is that the LOX reaction was not stoichiometric, since the desaturation degree in the different lipid classes was not the same (**Supplementary Table 1**). This was supported by an additional experiment when defined molecular species 36:6-MGDG and 36:6-PC were directly compared as substrates. Here the velocity of LiLOX towards the two substrates was almost the same, minimizing the influence of the head group as a factor (**Supplementary Figure 4**). Another factor that can explain the difference are amino acid residues located at the entrance of the LiLOX substrate channel. Since complex lipids penetrate the active site with their acyl chains first (Feussner and Wasternack, 1998), these amino acids may stabilize the headgroup of MGDG better than the one of PC, influencing thereby the overall turnover. Headgroups of phospholipids are rather small and may harbor two charges: the negatively charged phosphate group, and the positively charged amine (PE) or choline group (PC). The headgroup of MGDG is much bigger and not charged. Interestingly, the crystal structure of PaLOX has been obtained containing a PE molecule in the active site and two amino acid residues from PaLOX that are stabilizing the PE headgroup have been described (Garreta et al., 2013; Banthiya et al., 2016). One of them, R422 stabilizes the negatively charged phosphate group (**Figure 8A**, residue in dark gray, white label). When the modelled LiLOX structure (cyan) is superimposed to this protein (black), it can be observed that the adjacent α-helix number 2 on the opposite site of the entrance is not perfectly positioned, as it superposes with the PE substrate (**Figure 8A**, black vs. cyan helix in the center). In line with this observation the homologous α-helix number 2 from GmLOX1 was described to be very flexible opening thereby the active site when a substrate is present (Bradshaw and Gaffney, 2014). Nevertheless, in the crystal structure of PaLOX, the n-8 carbon of the acyl chain at sn_1_ of PE is located next to the iron atom, as it has been predicted to be the case for LiLOX (**Figure 8A**). Moreover, the five iron ligands in the predicted LiLOX structure were shown to be in concordance with those from PaLOX. Together this suggests that the model for LiLOX may be valid enough to derive predictions about interactions that could happen with a lipid headgroup at the entrance of the putative substrate channel of LiLOX. In contrast to PaLOX, no positively charged residues are observed in the entrance of the pocket of LiLOX, which could stabilize a negatively charged phosphate group. Instead, two asparagine and two glutamine residues were found on the corresponding α-helix of LiLOX, facing to the entrance of the channel in the model: N683, N687, Q690 and Q691 (**Figure 7A**, cyan residues). These amide groups may have a strong C=O dipole and a weaker N-H dipole, both being susceptible to act as hydrogen bond acceptors, possibly for one of the alcohol groups of the galactosyl headgroup of either MGDG or DGDG. Similarly, no acidic residue was observed on the central α-helix of LiLOX. Instead this helix is rather non-polar, being rich in leucine residues, but it has two serine residues S316 and S327 pointing towards the entrance of the pocket. Serines have a primary alcohol on their side chains, also being able to form polar interactions with a galactosyl headgroup. All the previous hypotheses correspond to interactions of LiLOX with a lipid species harboring 18 carbon acyl chains. In the case of a lipid species with an acyl chain of 16 carbons, a deeper penetration of the substrate will be required in order to align the n-8 carbon with the iron atom of LiLOX. In such a scenario, the headgroup can no longer be aligned with the residues S316, S327, N683, N687, Q690 and Q691. This less favorable penetration of the lipid in the substrate channel might explain why acyl chains of 16 carbons are oxidized at a slower rate than those acyl chains having 18 carbons, as revealed in the course of this study.

In the course of this study, lipidomics analysis was performed on *L. incisa* cultures, in order to find endogenous LiLOX products. The relative concentrations of plastidic lipids such as MGDG and DGDG were found to be significantly lowered after nitrogen starvation (**Supplementary Figure 7**). Nevertheless, no LiLOX products could be detected in the analysis. One factor seems to be that we failed to identify the final LiLOX oxidation products of MGDG *in vitro*. LiLOX was shown to be able to metabolize MGDG further than the initially formed hydroperoxides by two independent methods. This mirrors very likely the endogenous situation. In fact, the catalysis of MGDG turnover by LiLOX recorded *in vitro* clearly shows that conjugated dienes are formed during a first part of the reaction (**Supplementary Figure 5**). Yet this chromophore having a maximum absorbance at 234 nm is degraded during a second part of the reaction and the new products were not detected by their absorbance, they must have lost their chromophore and formation of a new chromophore was never observed. However, all yet known secondary reactions catalyzed by LOX should harbor a chromophore: Firstly, PpLOX1, soybean LOX, MpLOX7 and tomloxC were reported to have a lyase activity (Salch et al., 1995; Senger et al., 2005; Shen et al., 2014; Tawfik et al., 2017). Secondly, a number of LOXs were reported to harbor a peroxidase activity further oxidizing the hydroperoxide into a keto group (Kühn et al., 1991). Although both reactions would affect the absorbance of the hydroperoxide, both would still harbor a conjugated dienone having a maximum absorbance around 280 nm. Such an increase of the absorbance at 280 nm was barely observed during LiLOX reactions with MGDG rendering this reactions unlikely to occur. Thirdly, eLOX3, 12*R*-LOX and FoxLOX were also described to metabolize lipid hydroperoxides into epoxy alcohols (Yu et al., 2003; Brodhun et al., 2013). Although the corresponding epoxy alcohols have only one remaining double bond as a chromophore, they are almost not detectable by UV/vis-spectroscopy. However, these three enzymes were reported to produce epoxy alcohols always together with keto-fatty acids, and the formation of the latter was never observed with LiLOX. Since we could exclude a known reaction, a non-targeted analysis was performed by LC-MS, and the data were analyzed by automatic clustering using the software MarVis. As shown in **Figure 3**, all MGDG substrates and hydroperoxide products were detected by LC-MS. Besides them, none of the additional LOX-derived compounds mentioned above were detected after the LiLOX reaction with MGDG. Raw data analyzed by the untargeted MarVis workflow, highlighting compounds only at late stages of the reaction did not put reveal any new LiLOX products.

This study reports on the first LOX to be characterized from green algae: LiLOX, a plastidic 13*S*-LOX being able to use complex lipids as substrates, showing a preference towards MGDG. Contrary to *A. thaliana*, in which 13*S*-LOXs are known to catalyze the first step leading to the formation of JA-Ile, a similar pathway was yet not described in green algae (Wasternack and Feussner, 2018). However, some LOXs have also been reported to initiate the degradation of organelle membranes (Feussner et al., 1995; Feussner and Wasternack, 1998). Therefore, it is tempting to speculate that LiLOX may have a similar role in degrading plastidic membranes upon nitrogen starvation. LiLOX was shown to be overexpressed 6 fold under nitrogen starvation, a condition well described in green algae to lead to chloroplast degradation and the accumulation of TAG in lipid bodies (**Figure 1**; **Supplementary Figure 7**). Yet although TAG accumulation was first believed to be a storage mechanism, it is now also known to play a major role as electron sink when the energy from photosynthesis can no longer be used for central metabolism (Hu et al., 2008). Indeed, in *C. reinhardtii* the PGD1 protein was described to be involved in a pathway leading to TAG formation mostly from *de novo* acyl chain synthesis. The KO mutant *pgd1* was described to accumulate significantly less TAG, showed signs of chlorosis 7 days after nitrogen starvation and eventually cell death. As the artificial inhibition of the electron transport of photosynthesis prevented chlorosis and cell death in this mutant, the toxicity was imputed by the authors to reactive oxygen species formed by the accumulation of electrons in the thylakoid membrane (Li et al., 2012). Therefore, the function of LiLOX may be rather to target excess fatty acids to degradation for energy production by oxidizing them while they are bound to complex lipids during stress. Along with the TAG accumulation, nitrogen starvation lead to a change of color of *L. incisa* cultures from green to yellow, suggesting a degradation of chloroplasts. In addition, some LOXs have been reported to be involved in lipid body degradation in cucumber, barley, flax and sunflower during germination (Feussner et al., 1998). Interestingly, a patent application reported that in *C. reinhardtii* starch degradation 1 (*STD1*)-KO was reported to show overexpression of CrLOX transcript (6 log fold) suggesting a negative regulation of CrLOX from STD1 (Schulz-Raffelt et al., 2017). The galactolipid MGDG was measured to be degraded faster than in the WT after nitrogen starvation, although no effect was observed on DGDG. Oxidized MGDG species were also measured up to 18 fold higher than in the WT, after 6 days of nitrogen starvation. Finally, *STD1* mutant was also reported to accumulate more TAG than the WT under nitrogen starvation, effect abolished in the WT as well as in std1 after supplementation of the media with a LOX inhibitor.

## Conclusion

Altogether, the subcellular localization of LiLOX and its homology to other plastidic LOXs and its measured pH optimum suggest that LiLOX is located in the stroma of plastids. The fact that MGDGs are known to be the main lipid class in this organelle, as well as its overall degree of desaturation make this lipid a good candidate for LiLOX as natural substrate and suggests its involvement in chloroplast membrane remodeling. However, lipidomics analysis did not reveal so far an endogenous LiLOX product. Nevertheless, the end product of LiLOX reaction with MGDG is a key to understanding the role of this enzyme in *L. incisa* and should be considered a priority in the further analysis of LiLOX.

## Data availability statement

The original contributions presented in the study are included in the article/**Supplementary Material**. Further inquiries can be directed to the corresponding author.

## Author contributions

BD, KF, CH, EH and IF conceived and designed the experiments. BD, EH, CH, KZ and KF performed the experiments. BD, EH, KZ, CH, KF and IF analyzed and discussed the data, BD, KF, CH, EH and IF wrote the article. All authors contributed to the article and approved the submitted version.
