# Supplementary material for: Plastidic membrane lipids are oxidized by a lipoxygenase in Lobosphaera incisa
Source: Front Plant Sci. 2022 Dec 22;13:1102215. doi: 10.3389/fpls.2022.1102215 (PMC9813749; doi:10.3389/fpls.2022.1102215)
Supplement: Supplementary file 2 [file DataSheet_1.pdf]

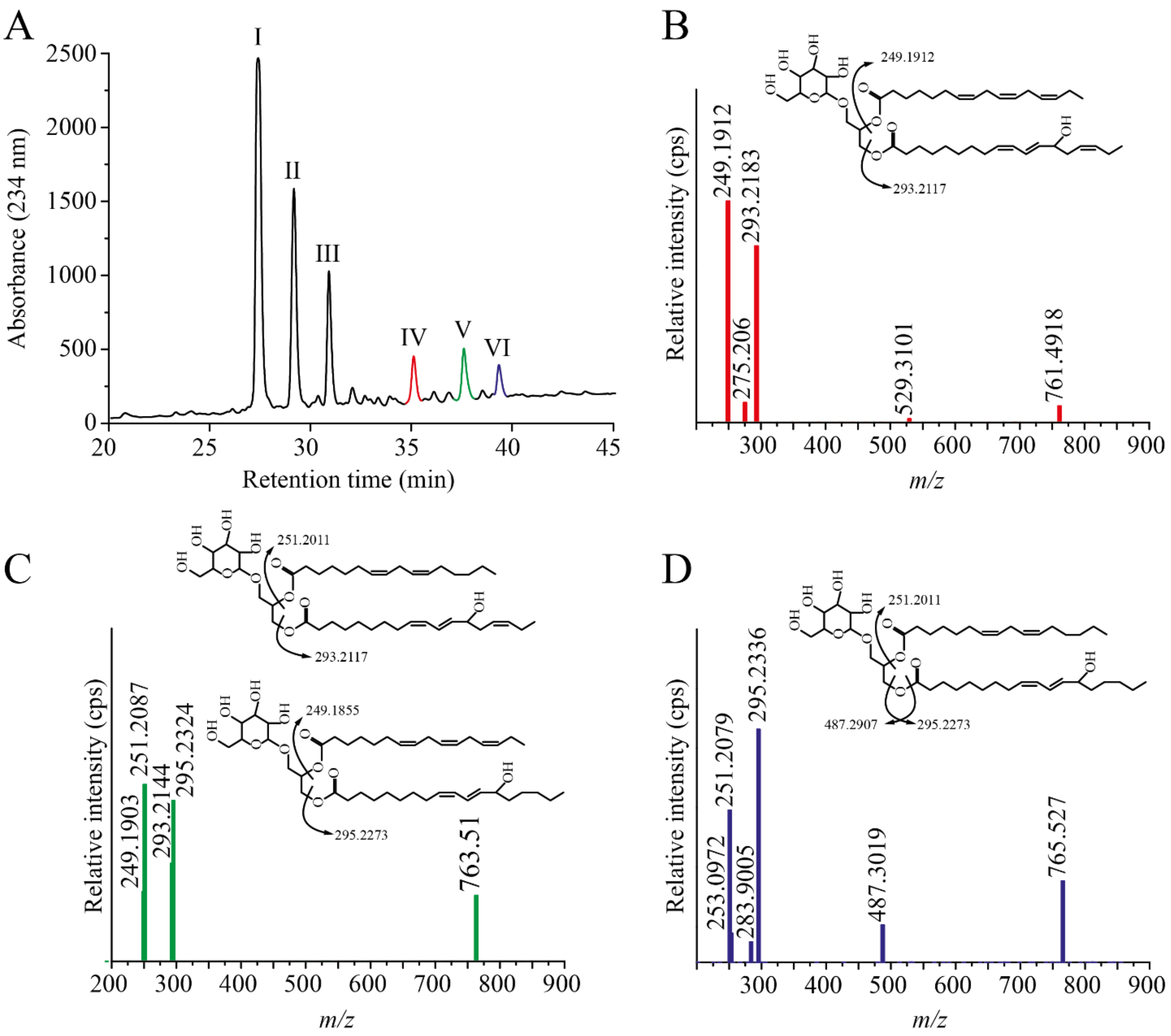

Supplementary Figure 1 Oxidation products of LiLOX with MGDG extracted from *L. incisa*. (**A**) RP-HPLC of all oxidation products after chemical reduction. Six peaks were identified, named I; II; III; IV; V and VI (same Figure as in 5A). (**B-D**) Fragmentation pattern of the purified features IV, V and VI obtained by LC-MSMS. The position of the hydroxyl groups on the chemical structures shown are were tentatively assigned. (**B**) Peak IV. (**C**) Peak V. (**D**) Peak VI.

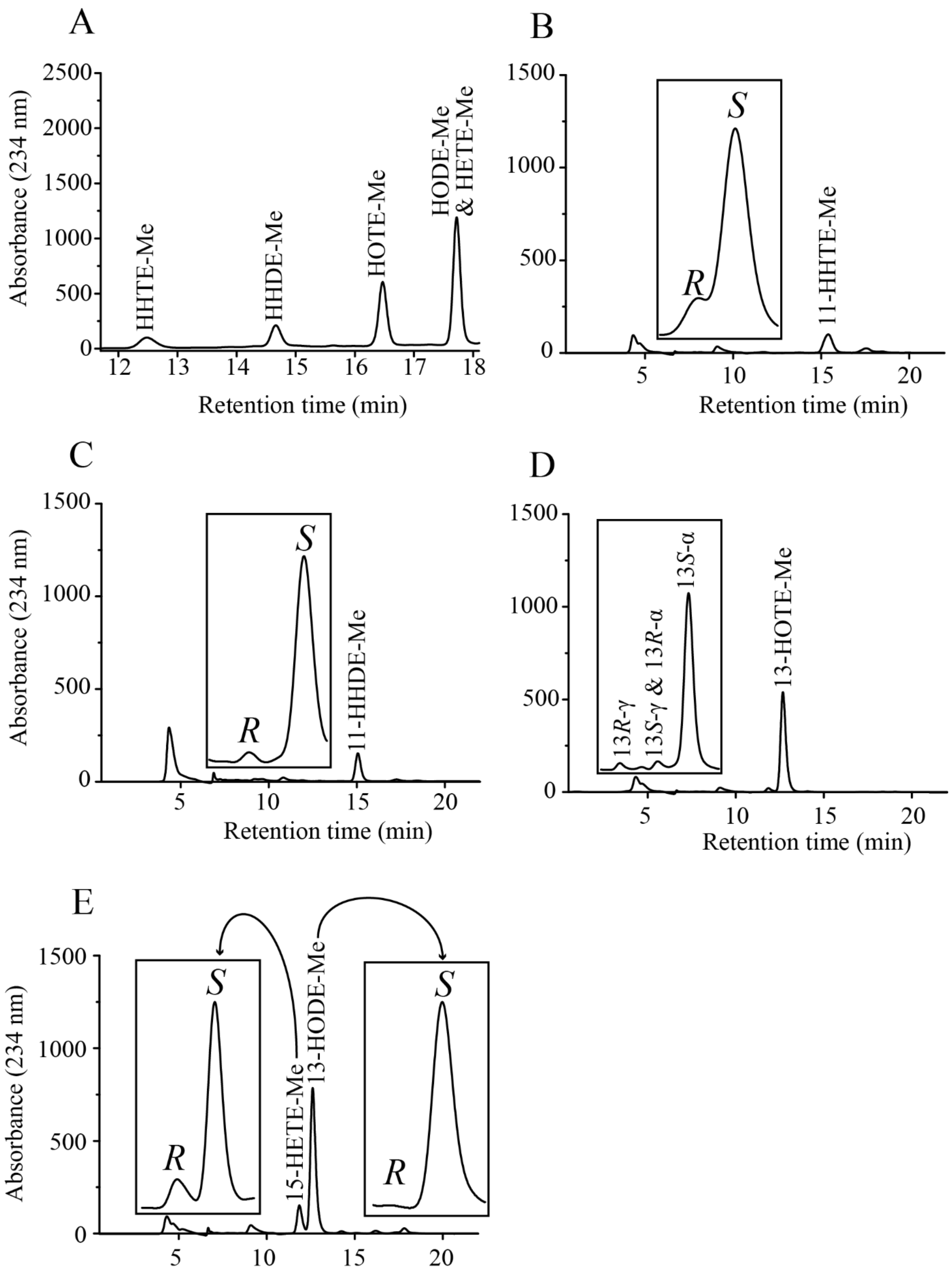

Supplementary Figure 2 Sequential HPLC analysis of oxidation products of LiLOX with DGDG extracted from *L. incisa*. (**A**) After transesterification of the acyl chains from the mixture of DGDG oxidation products, the oxidized fatty acid methyl esters were purified by RP-HPLC. (**B-E**) Sequential analysis of the oxidized fatty acid methyl ester purified as shown in (**A**) by SP-HPLC and CP-HPLC. The identity of the methyl-esters was confirmed by authentic standards. RP-HPLC and SP-HPLC chromatograms are representative of at least three measurements with independent enzyme preparations. CP-HPLC chromatograms are representative of single measurements.

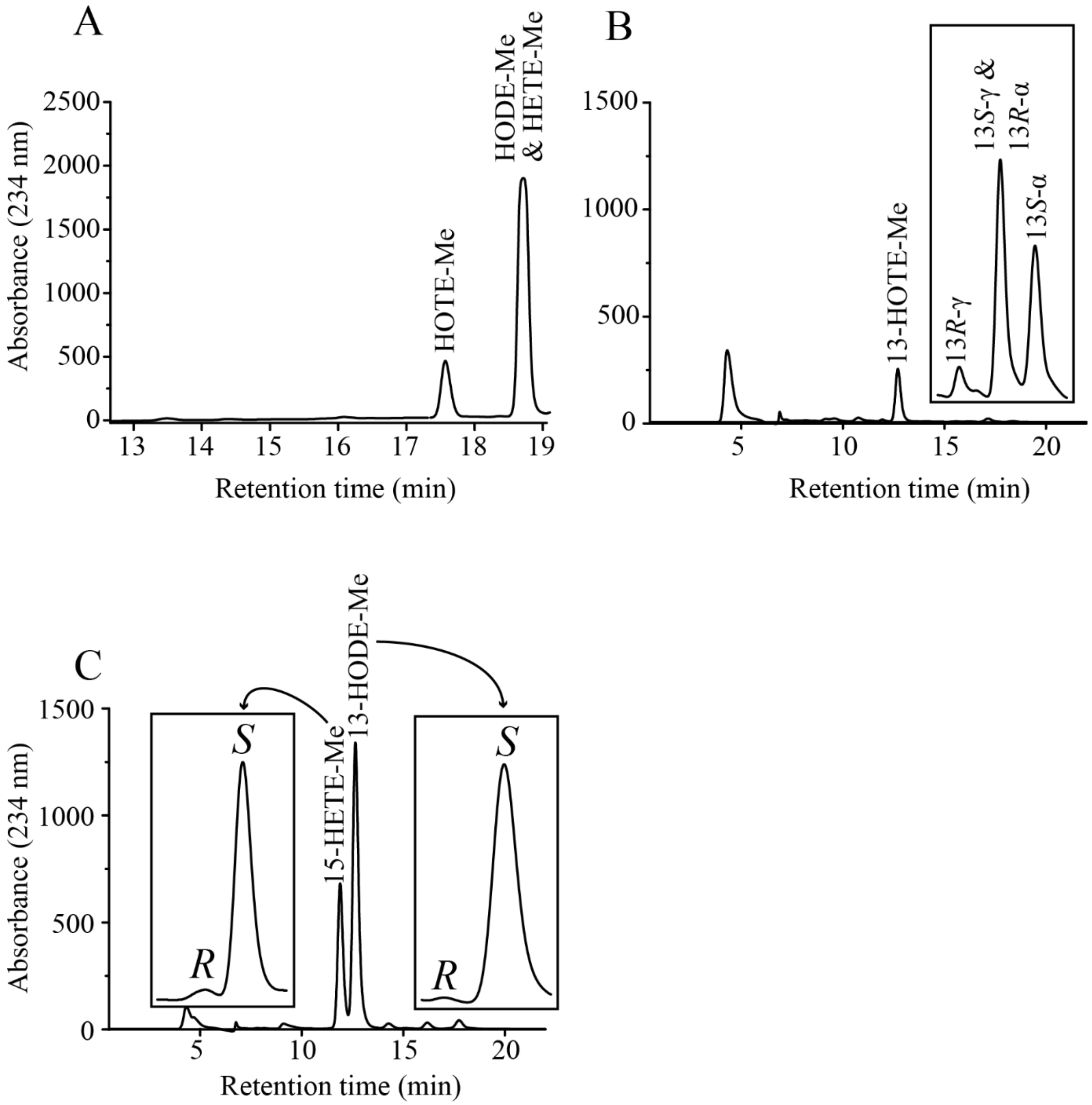

Supplementary Figure 3 Sequential HPLC analysis of oxidation products of LiLOX with PC extracted from *L. incisa*. (**A**) After transesterification of the acyl chains from the mixture of PC oxidation products, the oxidized fatty acid methyl esters were purified by RP-HPLC. (**B**-**C**) Sequential analysis of the oxidized fatty acid methyl esters purified as shown in (**A**) by SP-HPLC and CP-HPLC. The identity of the methyl esters was confirmed by authentic standards. RP-HPLC and SP-HPLC chromatograms are representative of at least three measurements with independent enzyme preparations. CP-HPLC chromatograms are representatives of single measurements.

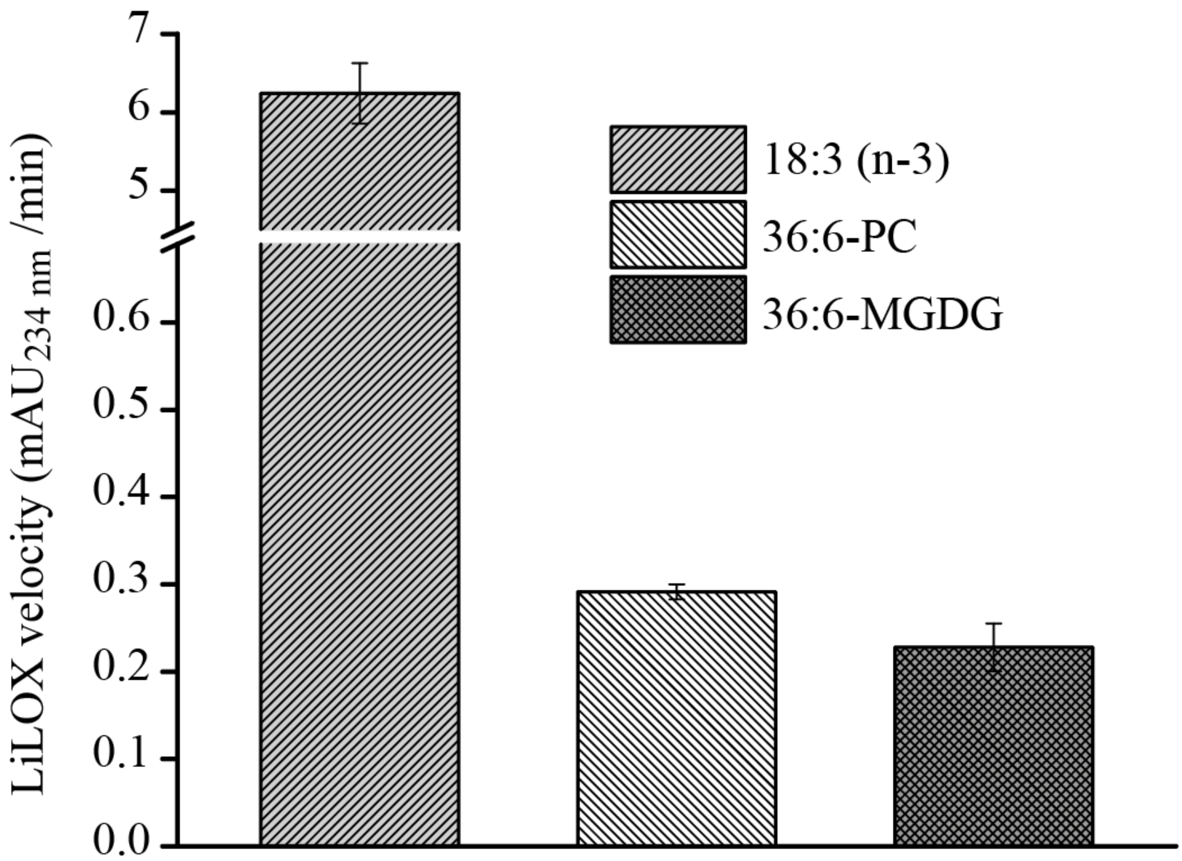

Supplementary Figure 4 Velocity of LiLOX WT with three different substrates: 18:3 (n-3); 18:3 (n-3)/18:3 (n-3)-MGDG; 18:3 (n-3)/18:3 (n-3)-PC. Each bar represents the average velocity of three measurements. Each measurement was performed with an independent enzyme preparation.

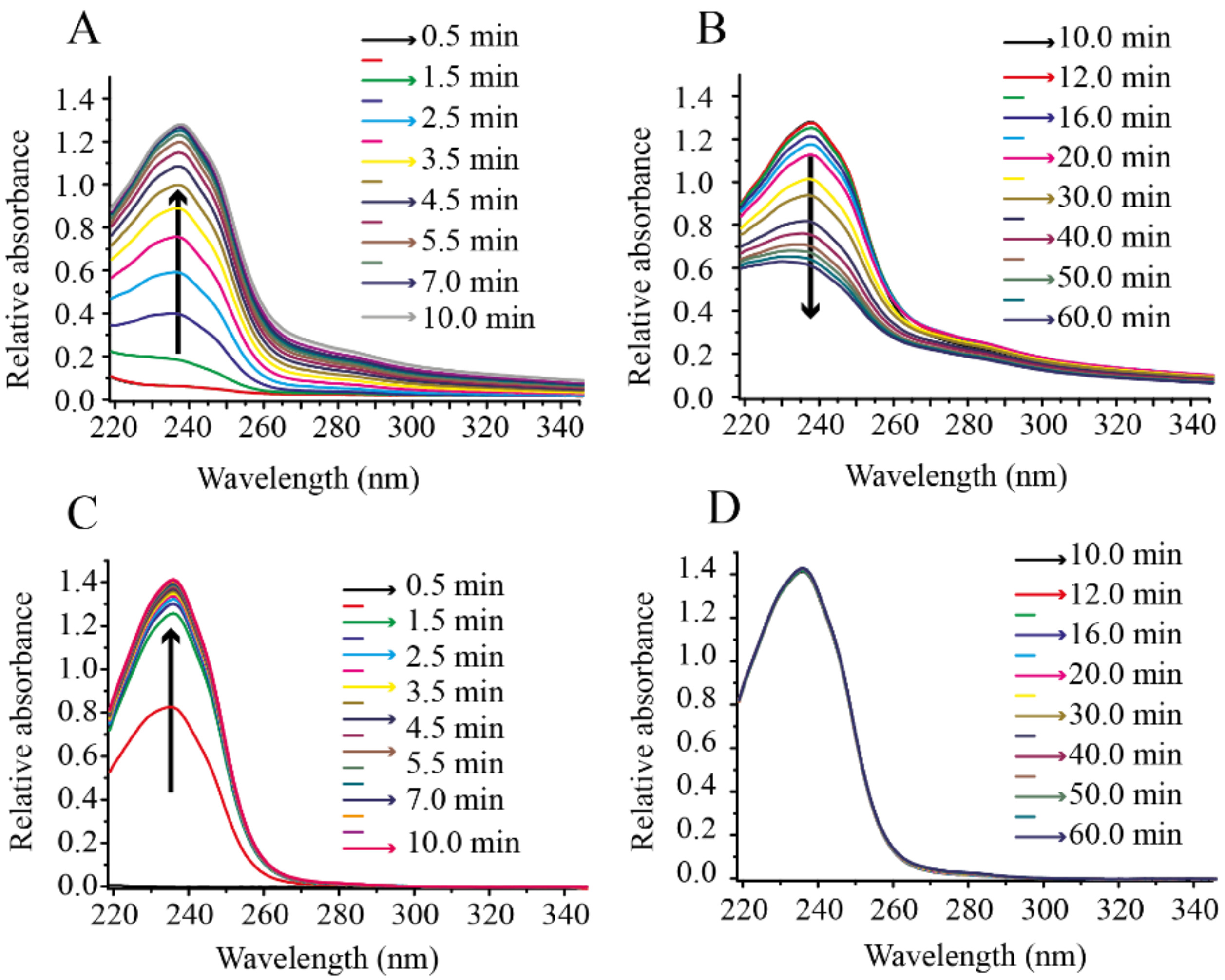

Supplementary Figure 5 Relative absorbance of LiLOX reaction during 1 hour. (**A**) LiLOX reaction with MGDG from 0 to 10 minutes. (**B**) LiLOX reaction with MGDG from 10 minutes to 60 minutes. (**C**) LiLOX reaction with 18:3 (n-3) from 0 to 10 minutes. (**D**) LiLOX reaction with 18:3 (n 3) from 10 minutes to 60 minutes. This measurement is representative for at least three measurements performed with independent enzyme preparations.

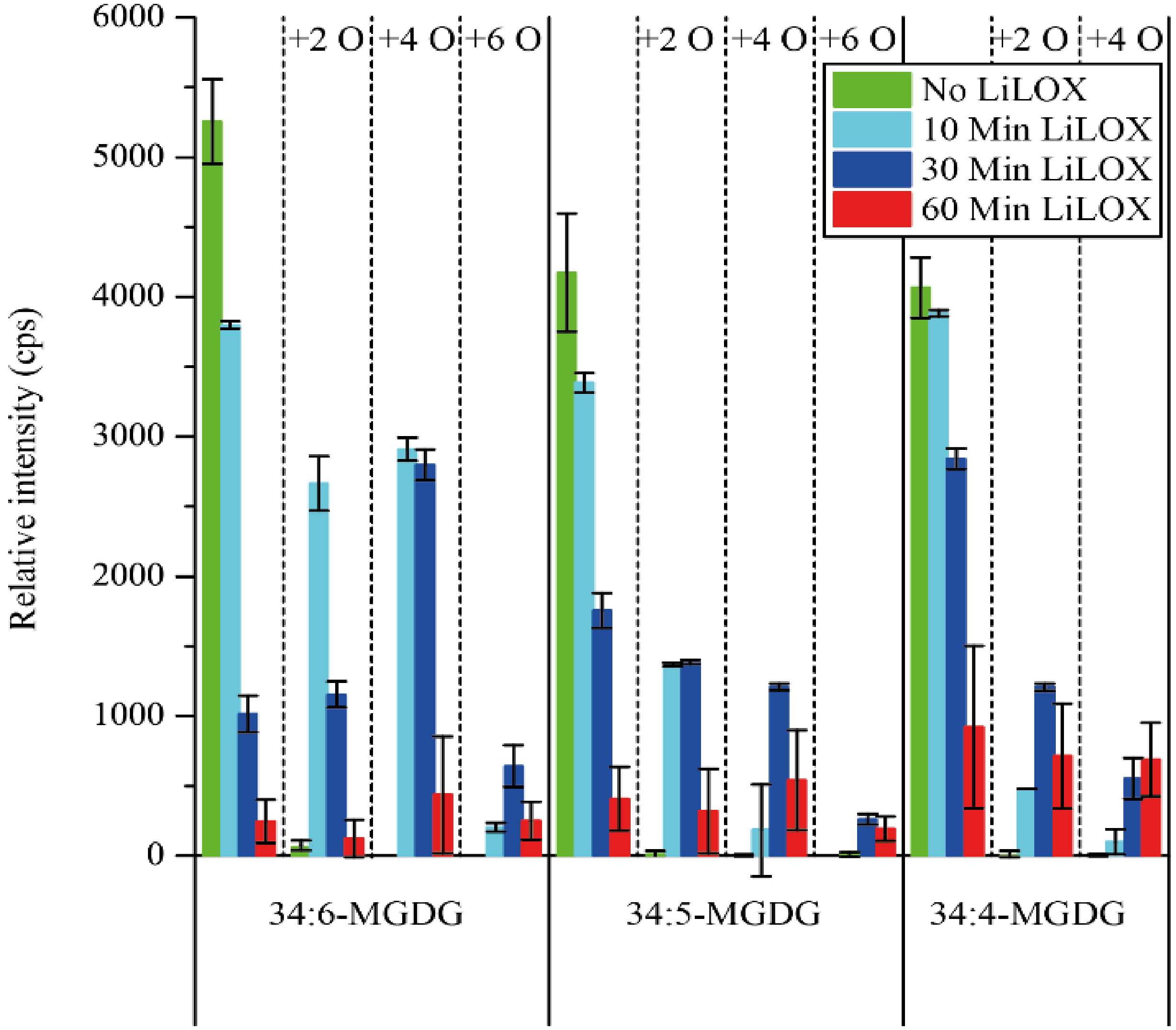

Supplementary Figure 6 Formation of LiLOX oxidation products with MGDG extracted from *L. incisa*. MGDG was solubilized in Bis-TRIS propane with 10 % (v/v) methanol. The reactions were started with the addition of 5 µL of LiLOX, stopped at a different time points (10 min, 30 min and 60 min) by addition of acetonitrile (1:1 v/v) and measured without further processing. Each bar represents MGDG molecules and their corresponding oxidized species with two (+2O), four (+4O) or six (+6O) additional oxygen atoms. Error bars represent the standard deviation of three experiments. Each experiment was performed with different enzyme preparations.

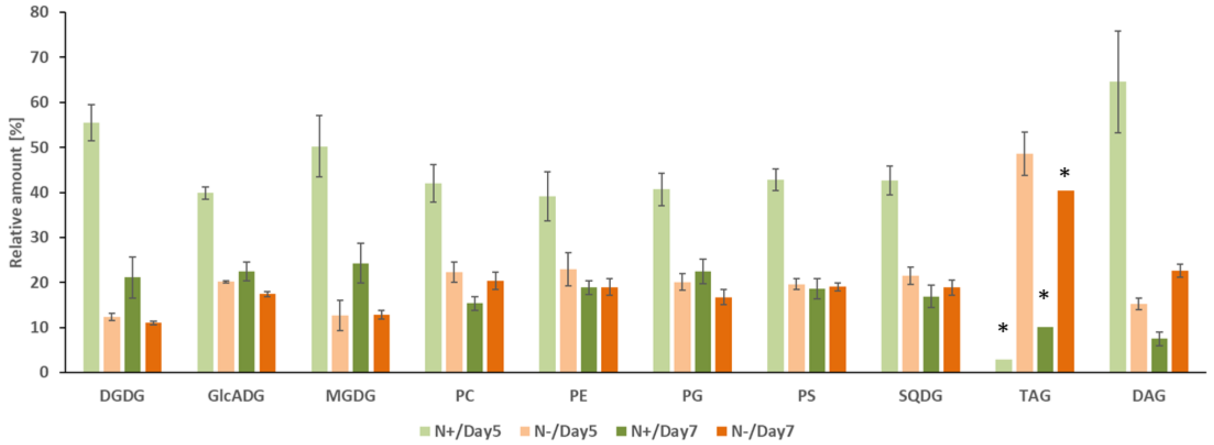

Supplementary Figure 7 Lipidomic analysis of *L. incisa* during nitrogen starvation. Cultures of *L. incisa* were grown in full BG11 media. The cells were then split in six different cultures, three with full BG11 media and three without any nitrogen source. Cells were collected at different time points, and lipidomic analysis was performed by LC-MS/MS. The graph shows the difference of lipid profiles after nitrogen starvation compared to growth in full media. The peak area of all detected molecular species of each lipid class were summed and then each was converted to relative amounts across all four conditions. The corresponding data are shown in Supplementary Table 1. Each bar represents the average of three samples grown in parallel. Error bars represent the standard deviation of the three samples grown in parallel. *Average of two measurements, no error bar.

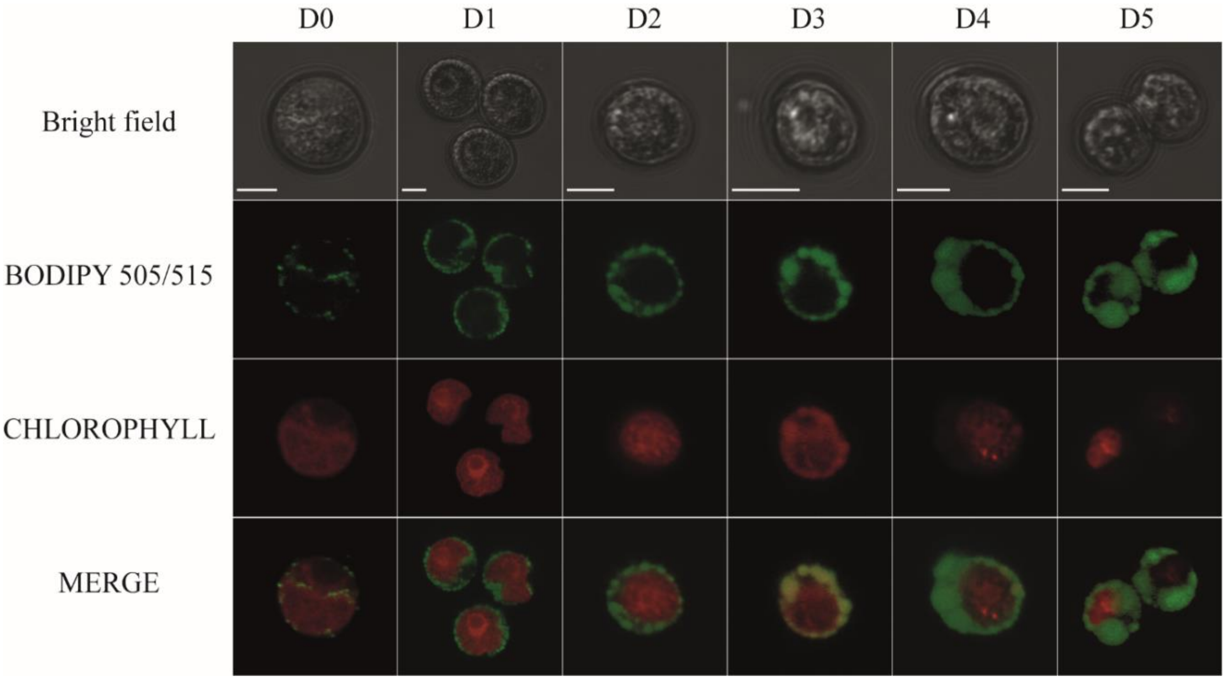

Supplementary Figure 8 Fluorescent microscopy of *L. incisa* cells during nitrogen starvation. BODYPY 505/515 was used to label lipid droplets, shown in green. Auto-fluorescence from chlorophyll reveals chloroplasts, shown in red.
